# High- versus Low-Spin Ni^2+^ in Elongated Octahedral Environments: Sr_2_NiO_2_Cu_2_Se_2_, Sr_2_NiO_2_Cu_2_S_2_, and Sr_2_NiO_2_Cu_2_(Se_1–*x*_S_*x*_)_2_

**DOI:** 10.1021/acs.chemmater.2c02002

**Published:** 2022-10-18

**Authors:** Robert D. Smyth, Jack N. Blandy, Ziyu Yu, Shuai Liu, Craig V. Topping, Simon J. Cassidy, Catherine F. Smura, Daniel N. Woodruff, Pascal Manuel, Craig L. Bull, Nicholas P. Funnell, Christopher J. Ridley, John E. McGrady, Simon J. Clarke

**Affiliations:** † Inorganic Chemistry Laboratory, Department of Chemistry, University of Oxford, South Parks Road, OxfordOX1 3QR, U.K.; ‡ Diamond Light Source Ltd., Harwell Science and Innovation Campus, DidcotOX11 0DE, U.K.; § College of Chemistry and Chemical Engineering, Anhui University, Hefei230601, People’s Republic of China; ∥ Clarendon Laboratory, Department of Physics, University of Oxford, Parks Road, OxfordOX1 3PU, U.K.; ⊥ ISIS Facility, Rutherford Appleton Laboratory, Harwell Oxford, DidcotOX1 10QX, U.K.; # School of Chemistry, The University of Edinburgh, King’s Buildings, David Brewster Road, EdinburghEH9 3FJ, U.K.

## Abstract

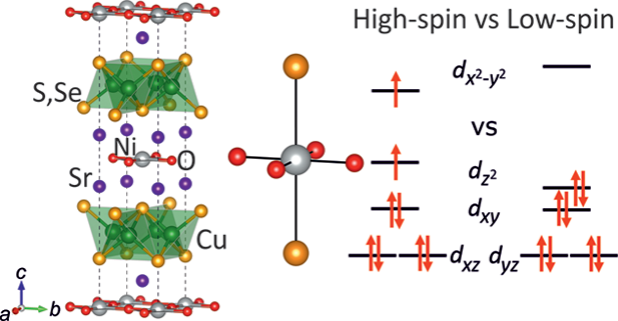

Sr_2_NiO_2_Cu_2_Se_2_, comprising alternating [Sr_2_NiO_2_]^2+^ and [Cu_2_Se_2_]^2–^ layers, is reported. Powder neutron diffraction shows that the Ni^2+^ ions, which are in a highly elongated NiO_4_Se_2_ environment with D_4*h*_ symmetry, adopt a high-spin configuration and carry localized magnetic moments which order antiferromagnetically below ∼160 K in a √2*a* × √2*a* × 2*c* expansion of the nuclear cell with an ordered moment of 1.31(2) μ_B_ per Ni^2+^ ion. The adoption of the high-spin configuration for this *d*^8^ cation in a pseudo-square-planar ligand field is supported by consideration of the experimental bond lengths and the results of density functional theory (DFT) calculations. This is in contrast to the sulfide analogue Sr_2_NiO_2_Cu_2_S_2_, which, according to both experiment and DFT calculations, has a much more elongated ligand field, more consistent with the low-spin configuration commonly found for square-planar Ni^2+^, and accordingly, there is no evidence for magnetic moment on the Ni^2+^ ions. Examination of the solid solution Sr_2_NiO_2_Cu_2_(Se_1–*x*_S_*x*_)_2_ shows direct evidence from the evolution of the crystal structure and the magnetic ordering for the transition from high-spin selenide-rich compounds to low-spin sulfide-rich compounds as a function of composition. Compression of Sr_2_NiO_2_Cu_2_Se_2_ up to 7.2 GPa does not show any structural signature of a change in the spin state. Consideration of the experimental and computed Ni^2+^ coordination environments and their subtle changes as a function of temperature, in addition to transitions evident in the transport properties and magnetic susceptibilities in the end members, Sr_2_NiO_2_Cu_2_Se_2_ and Sr_2_NiO_2_Cu_2_S_2_, suggest that simple high-spin and low-spin models for Ni^2+^ may not be entirely appropriate and point to further complexities in these compounds.

## Introduction

**Figure 1:**
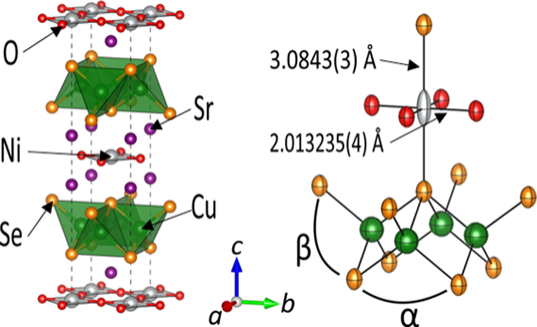
Crystal structure^4^ of Sr_2_NiO_2_Cu_2_Se_2_, highlighting the coordination environment of the Ni and Cu sites. Anisotropic displacement parameters from the combined refinement against X-ray and powder neutron diffraction data are shown at 99% probability. It should be noted that the Ni–O distance equals half the basal lattice parameter, so it is determined with very high precision.

Layered oxide chalcogenides of composition *A*_2_*M*O_2_*M′*_2_*Ch*_2_ (*A* = Sr, Ba; *M* = Mn, Co, Ni, Cu, Zn; *M′* = Cu, Ag; and *Ch* = S, Se) were first described by Zhu and Hor in 1997.^1,2^ This series adopts a crystal structure similar to that first described for Sr_2_Mn_3_Sb_2_O_2_,^3^ as shown in Figure 1. The oxide and chalcogenide (or pnictide) anions segregate into distinct layers, and transition or main group cations may be incorporated into elongated octahedral *M*O_4_*Ch*_2_ sites in the oxide layers or slightly distorted *M′Ch*_4_ tetrahedral sites in the chalcogenide layers. The distribution of the metal cations is normally determined by their chemical requirements. Commonly, mid-to-late-series first-row transition metal ions occupy the sites in the oxide layers while the more chalcophilic coinage metals occupy the sites in the chalcogenide layers.

These compounds display a rich array of properties associated with the identity of the transition metals. The highly elongated octahedral coordination environment enforced by the crystal structure offers a counterpoint to the more regular octahedral coordination generally found in pure oxide analogues such as perovskite and Ruddlesden–Popper oxide phases, and the possibility of nonstoichiometry on the coinage metal site in the chalcogenide layers leads to the possibility of composition and property-tuning using soft chemistry.^5^ Furthermore, the synthesis under anaerobic conditions along with the presence of heavier chalcogens, rather than solely oxygen, often leads to the stabilization of lower transition metal oxidation states than those that are generally found in ternary oxides such as perovskites. For example, Mn^2+/3+^ mixed valence is common in these oxide chalcogenides in contrast to the Mn^3+/4+^ mixed valence, which is important in oxide chemistry.^6−9^

Sr_2_NiO_2_Cu_2_S_2_ was first reported by Otzschi et al.,^10^ albeit with relatively low purity because of the difficulty of avoiding the competing decomposition by reduction of Ni^2+^ and Cu^+^ to the metals with the corresponding oxidation of S^2–^ to form SO_4_^2–^ at elevated temperatures. Clarke et al.^11^ reported a low-temperature synthesis using an alkali-metal halide flux, which led to a much purer sample, from which it was established that Sr_2_NiO_2_Cu_2_S_2_ displays no long-range magnetic ordering on the basis that there were no measurable additional Bragg peaks in the low-temperature neutron diffractogram. This is consistent with a low-spin Ni^2+^ ion in the elongated octahedral (i.e., almost square-planar) environment, in line with molecular compounds containing Ni^2+^ in a square-planar ligand field. Transitions observed in the magnetic susceptibility measurements and an increase in the elongation of the NiO_4_S_2_ octahedron on cooling appeared qualitatively consistent with a “high-spin to low-spin” transition for the Ni^2+^ ion on cooling, but the changes in the Ni–S/Ni–O ratio were approximately an order of magnitude smaller than what would be expected for such a change in the spin state.

Recently, Matsumoto et al. reported that the isostructural analogues *A*_2_NiO_2_Ag_2_Se_2_ (*A* = Sr, Ba) with the larger coinage metal ion Ag^+^ in the chalcogenide layer were attainable using high-pressure synthesis.^12^ They showed, using a combination of experimental and computational methods that, in contrast with Sr_2_NiO_2_Cu_2_S_2_, these compounds had the Ni^2+^ ion in a high-spin configuration, which they ascribed to the reduced ligand field strength resulting from the longer Ni–O bonds enforced by the accommodation of the other larger ions and the weak contribution to the Ni bond valence from the apical selenide anions.

Here, we focus on a new compound, Sr_2_NiO_2_Cu_2_Se_2_, and show from the analysis of the crystal structure and the physical and magnetic properties backed up by density functional theory (DFT) calculations that this compound and the sulfide analogue essentially lie close to, but on opposite sides of, the high-spin/low-spin boundary in this system. We traverse this boundary as a function of composition with the solid solution Sr_2_NiO_2_Cu_2_(Se_1–*x*_S_*x*_)_2_.

## Experimental Section

### Synthesis

Because of some of the reagents being air-sensitive, all manipulations of solids were performed in an argon-filled glovebox (Glovebox Technology Ltd., with O_2_ and H_2_O contents typically below 1 ppm). Samples of Sr_2_NiO_2_Cu_2_Se_2_ were synthesized from a mixture of SrO (obtained by thermal decomposition under dynamic vacuum of SrCO_3_ (Alfa 99.994%), at initially 840 °C with a final treatment at 1100 °C), Ni (Alfa 99.996%), Cu (Alfa 99+%), and Se (Alfa 99.999%) in the molar ratio 2:1:2:2. The powders were ground together, pelletized at 0.4 GPa, and the pellet was loaded into an alumina crucible and sealed in an evacuated silica ampule (19 mm o.d.). This assembly was then heated in a resistance furnace. The purest sample, used for most of this work, was synthesized on a 5 g scale by heating the pelletized reagents to 400 °C at 10 °C min^–1^, maintaining this temperature for 15 h, then raising the temperature to 850 °C for a further 24 h, followed by furnace cooling. After regrinding, the repelletized sample was then placed directly in a furnace at 850 °C, and after 16 h, the tube was quenched in ice/water. Attempts to synthesize Sr_2_NiO_2_Cu_2_Se_2_ at temperatures lower than the 850 °C reported here resulted in significant amounts of impurities and different lattice parameters for the main phase compared with those found in the purest products. This was partially due to Ni/Cu disorder between the oxide and chalcogenide layers (see the Discussion and Conclusions section and the Supporting Information). For this reason, the quenching step was necessary. Quenching from 950 °C also gave high-purity products.

The selenide-rich members of the solid solution Sr_2_NiO_2_Cu_2_(Se_1–*x*_S_*x*_)_2_ with 0.125 ≤ *x* ≤ 0.5 were synthesized in a similar way to Sr_2_NiO_2_Cu_2_Se_2_ by grinding together stoichiometric amounts of SrO, Ni (Alfa 99.996%), Cu (Alfa 99+ %), and an appropriate amount of S (Alfa 99.999%) or Se (Alfa 99.999%). Pellets were prepared as described above, placed in alumina crucibles, and sealed in evacuated silica ampules with the sample-containing ends of the ampules submerged in liquid N_2_ during flame sealing to avoid the vaporization of elemental S. These samples were then preheated at 400 °C, ramping up at 10 °C min^–1^, for 12 h to avoid the vapor pressure inside the silica tube getting too high while the chalcogen reacted, before final heating at 950 °C for 16 h, again ramping at 10 °C min^–1^ with a similar quenching protocol to the pure selenide used at the end of the heating cycle. For the more S-rich compounds (0.625 ≤ *x* ≤ 0.875), using the high-temperature approach led to significant amounts of Cu_*x*_Ni_*y*_ and SrSO_4_ impurities (see Figure S1), and so, a synthetic route similar to that used for Sr_2_NiO_2_Cu_2_S_2_^11^ was used, whereby a low-melting alkali-metal halide flux consisting of 0.56:0.44 CsI:NaI was employed. This eutectic mixture with a melting point of 435 °C^13^ was prepared using “ultra-dry” CsI (Alfa 99.998%) and NaI (Alfa 99.9% predried at 300 °C for 12 h). The iodide mixture was then ground together with the reaction mixture, and the mixture was heated in an alumina crucible sealed in an evacuated silica ampule at 500 °C for 15 h. This was repeated until no further change was observed in the diffraction pattern, which was usually after three heating cycles. Using a low-temperature synthetic route encourages the formation of the S-rich solid solution phases without leading to decomposition of the target phase.

### Diffraction Measurements

Initial structural characterization was carried out by powder X-ray diffraction (PXRD) using a PANalytical Empyrean diffractometer operating in the Bragg–Brentano geometry with a Ge(111) monochromator to select only the Cu*K*_α1_ radiation. Detailed structural characterization of Sr_2_NiO_2_Cu_2_Se_2_ was undertaken on the high-resolution synchrotron X-ray powder diffractometer ID22^14^ at the ESRF, France, and the solid solution Sr_2_NiO_2_Cu_2_(Se_1–*x*_S_*x*_)_2_ samples were measured on the I11 beamline at the Diamond Light Source, UK.^15^ Samples were prepared for such measurements by mixing the sample with amorphous boron to minimize absorption and preferred orientation effects and then loading the mixture into borosilicate capillaries (0.5 or 0.7 mm o.d.) under argon. Powder neutron diffraction (PND) measurements to probe changes to the crystal structure as a function of temperature, to characterize the magnetic long-range order, as well as to assess possible Ni/Cu disorder, were performed on two portions of the same Sr_2_NiO_2_Cu_2_Se_2_ sample on the time-of-flight diffractometers HRPD^16^ and OSIRIS^17^ at the ISIS Pulsed Neutron Source, UK. HRPD is optimized for high-resolution diffraction measurements at low *d*-spacings, while OSIRIS is optimized for the measurement of data at long *d*-spacings, so it is better suited to probe magnetic ordering. On HRPD, 2.0 g of Sr_2_NiO_2_Cu_2_Se_2_ was loaded into a 2 mm deep rectangular aluminum cell with 40 mm × 40 mm vanadium windows (a “slab can”) with an integral heating element and thermocouple for efficient control of temperature within a cold cryostat. On OSIRIS, ≈ 2.5 g of the same sample was contained in a cylindrical vanadium can. Both types of container were sealed with indium gaskets. Measurements as a function of applied pressure were performed on Sr_2_NiO_2_Cu_2_Se_2_ using the PEARL^18^ diffractometer at the ISIS facility. PEARL is a high-flux, medium resolution diffractometer dedicated to high-pressure measurements. Ground Sr_2_NiO_2_Cu_2_Se_2_ was loaded into an encapsulated gasket machined from null-scattering TiZr alloy.^19^ A methanol/ethanol mixture (4:1) was used as the pressure-transmitting medium to provide quasi-hydrostatic conditions^20^ and was added to the sample chamber along with a small piece of elemental lead to act as an internal pressure marker for which the equation of state (EoS) is well known.^21^ The gasket assembly was secured between a pair of single toroidal profile anvils^22^ made from zirconia/alumina for measurements in the low-pressure region and sintered diamond for analysis at elevated pressures. This setup was then placed in a V3 Paris–Edinburgh press^23^ and a sealing load was applied. Diffraction patterns were obtained at the 2θ = 90° detector which allowed for *d*-spacings between 0.5 and 4.1 Å to be accessed. The data were subsequently corrected for anvil attenuation using the Mantid software.^24^ Ambient pressure and variable temperature PND measurements on a series of 1 g samples from the solid solution Sr_2_NiO_2_Cu_2_(Se_1–*x*_S_*x*_)_2_ (0.125 ≤ *x* ≤ 0.875) were performed using the WISH diffractometer at the ISIS facility.^25^ This instrument is optimized for high count rates at long *d-*spacings with high resolution and is particularly well suited to measurements of magnetic ordering, while also offering short *d*-spacing data adequate for the refinement of the nuclear structure. Samples were contained in 6 mm vanadium cylinders sealed with indium gaskets, and a closed-cycle refrigerator was used to cool the samples. Rietveld refinement against both PND and PXRD data were conducted using the TOPAS Academic Version 6 software.^26^ The magnetic structures of the Sr_2_NiO_2_Cu_2_(Se_1–*x*_S_*x*_)_2_ (0 ≤ *x* ≤ 0.3125) samples were deduced using ISODISTORT^27^ in conjunction with TOPAS Academic.

### Magnetometry

Magnetic susceptibilities were measured using Quantum Design MPMS-XL and MPMS-3 SQUID magnetometers. Approximately 30 mg batches of powder were contained in gelatin capsules. A measurement of magnetization vs field at 300 K revealed the presence of a small ferromagnetic impurity in the sample of Sr_2_NiO_2_Cu_2_Se_2_ (quenched from 850 °C) used for the structural measurements that presumably arose from trace amounts of elemental Ni or Cu_*x*_Ni_*y*_ alloy; therefore, in order to obtain reliable values for the magnetic susceptibility, the measurement was carried out during warming in fields of 30 and 40 kOe. These fields are both significantly larger than the saturation field of the ferromagnetic impurity, so a subtraction could be performed to obtain a good estimation of the magnetic susceptibility of Sr_2_NiO_2_Cu_2_Se_2_ in an “effective” 10 kOe field. In a subsequent sample, quenching from 950 °C, as used for the Se-rich members of the solid solution, seemed to eliminate any trace of the ferromagnetic impurities (confirming that they were extrinsic), giving a linear magnetization vs field plot (Figure S2).

### Resistivity Measurements

The DC resistivity of a sintered pellet of Sr_2_NiO_2_Cu_2_Se_2_ was measured using the four-probe method. Four copper (0.1 mm diameter; Alfa Aesar 99.9985%) wires were attached to the sintered pellet using silver paste. Measurements were made in the temperature range 15 ≤ *T* (K) ≤ 300 with the sample contained in a closed-cycle refrigerator system (AS Scientific Products Ltd) and controlled using an Oxford Instruments ITC-4 temperature controller. A current of 10 mA was applied using a Time Electronics 1024 DC current calibrator, and the voltage was measured using a Hewlett Packard HP3478A multimeter. Measurements at 1.0, 5, and 10 mA gave consistent results. Further measurements were not conducted on the other samples because consistent high-temperature powder sintering conditions for the solid solution were not possible due to the fact that the S-rich phases decompose above 500 °C. It is likely that flux growth of single crystals would offer the best chance to make comparative transport measurements across the whole series.

### Heat Capacity Measurements

The heat capacity of a cold-pressed pellet of Sr_2_NiO_2_Cu_2_Se_2_ (quenched from 850 °C) was measured as a function of temperature using a Quantum Design Physical Property Measurement System (PPMS) in static magnetic fields of 0 and 110 kOe.

### Computation

DFT calculations on the series Sr_2_*M*O_2_Cu_2_*Ch*_2_ (*M* = Co, Ni, Cu, Zn; *Ch* = S, Se) were carried out using the VASP software package,^28,29^ version 6.2.1, with the PBE functional^30^ and a plane-wave cut-off of 850 eV. A √2*a* × √2*a* × *c* expansion of the unit cell containing two *M*^2+^ centers per layer was used throughout, and the tetragonal Brillouin zone was sampled on a 4 × 4 × 2 Γ-centered grid.^31^ A finer 10 × 10 × 1 Γ-centered grid was used to generate the density of states (DOS). The effect of the core electrons was incorporated using projector augmented wave (PAW) potentials,^32^ and a Hubbard *U*_eff_ value^33^ of 2 eV was applied to the transition metal cations in the oxide layer. The DFT-D3 method was introduced for the van der Waals forces correction.^34,35^

## Results and Discussion

### Synthesis

Initial attempts to synthesize Sr_2_NiO_2_Cu_2_Se_2_ at the low temperatures (500–650 °C) required for Sr_2_NiO_2_Cu_2_S_2_ resulted in samples with large amounts of SrO, SrSe and Cu_1–*x*_Ni_*x*_ impurities. The lattice parameters of the main phase were also highly variable, consistent with the formation of Sr_2_Ni_1–*x*_Cu_*x*_O_2_Cu_2_Se_2_, with significant amounts of Cu in the oxide layer (see Discussion and Figure S3). This led to the use of the higher synthesis temperature of 850 °C and the quenching protocol which resulted in a much purer stoichiometric product used for a more detailed analysis. While this method was also successful for the Se-rich members of the solid solution, it was found that, like in the case of Sr_2_NiO_2_Cu_2_S_2_, a low-temperature route using a low-melting alkali-metal halide flux of 0.56:0.44 CsI:NaI was required for the S-rich compounds Sr_2_NiO_2_Cu_2_(Se_1–*x*_S_*x*_)_2_ where *x* ≥ 0.625, with repeated heating cycles at 500 °C.

### Crystal Structure of Sr_2_NiO_2_Cu_2_Se_2_

**Figure 2:**
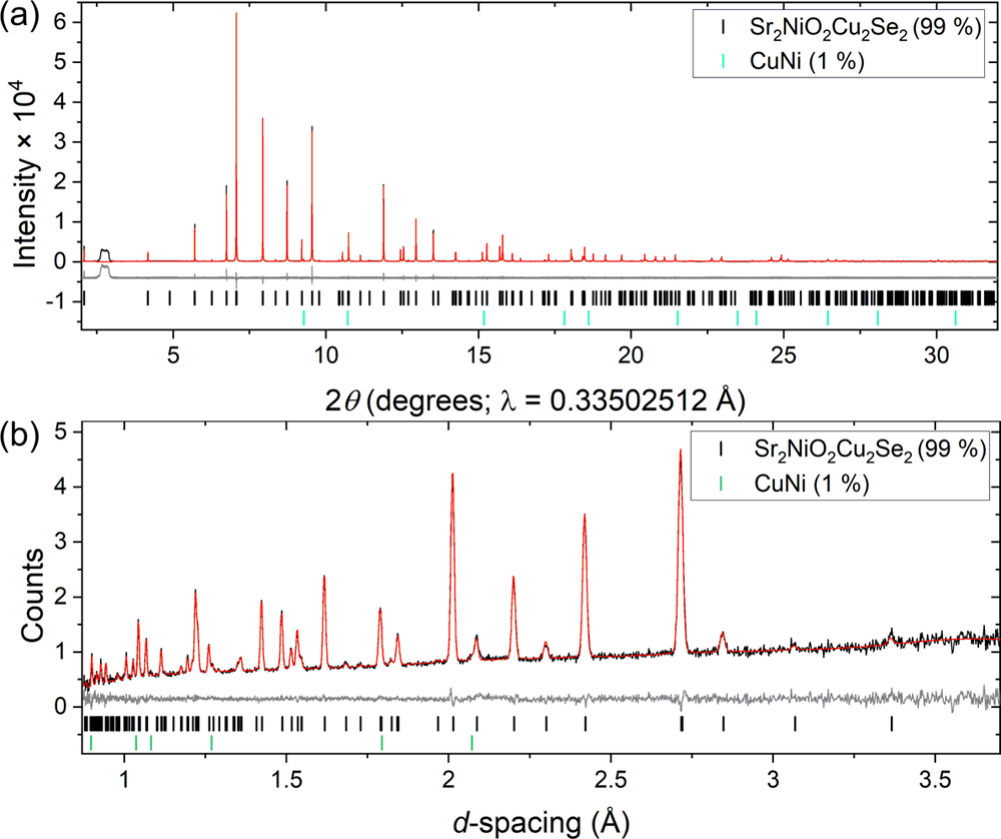
Rietveld refinements for Sr_2_NiO_2_Cu_2_Se_2_ at room temperature. (a) X-ray data from ID22, the unindexed feature at ≈ 3° is an artifact of the sample environment. (b) PND data from the 90° bank of HRPD. Data (black), calculated (red), difference (gray), and reflection positions are shown. *R*_wp_ = 6.968%; χ^2^ = 1.459.

The structure and composition of Sr_2_NiO_2_Cu_2_Se_2_ were probed using combined refinement against synchrotron PXRD (ID22) and high-resolution PND measurements at room temperature (RT). These measurements showed that at RT, Sr_2_NiO_2_Cu_2_Se_2_ adopts the same structure as Sr_2_NiO_2_Cu_2_S_2_ in the *I*4/*mmm* space group with all sites fully occupied within the uncertainties of the refinements. A small amount (0.4% by mass) of a Cu_1–*x*_Ni_*x*_ impurity with *x* ≈ 0.2 according to the lattice parameters^36^ was evident in the data from ID22 in the large sample used for PND measurements. Attempts to model the Ni site in Sr_2_NiO_2_Cu_2_Se_2_ as a disordered site containing both Cu and Ni in the PND data resulted in a refined Cu occupancy on this site of 0.00(2), suggesting that the site within the oxide layer consists solely of Ni when the optimized synthetic conditions are used. The coherent neutron scattering lengths of Ni and Cu (10.3 and 7.718 fm, respectively)^37^ are sufficiently different that this question of disorder would be expected to be resolved using PND. The Rietveld plots against the synchrotron PXRD data and PND data are shown in Figures 2 and S4. The refined structural parameters are given in Table 1 and depicted in Figure 1.

**Table 1:** Refined^a^ RT Structural Parameters for Sr_2_NiO_2_Cu_2_Se_2_^b^

atom	site	*x*	*y*	*z*	*U*_11_ (Å^2^)	*U*_22_ (Å^2^)	*U*_33_ (Å^2^)
Sr	4*e*	0	0	0.41257(2)	0.0060(1)	= *U*_11_	0.0076(2)
Ni	2*a*	0	0	0	0.0042(2)	= *U*_11_	0.0256(5)
O	4*d*	0	1/2	0	0.0084(9)	0.0066(9)	0.013(1)
Cu	4*c*	0	1/2	1/4	0.0181(2)	= *U*_11_	0.0187(3)
Se	4*e*	0	0	0.16757(2)	0.0075(2)	= *U*_11_	0.0129(3)

a Combined refinement against synchrotron PXRD (ID22) and PND (HRPD) data.

b Space group: *I*4/*mmm. a* = *b* = 4.026470(8) Å; *c* = 18.40612(4) Å; Volume = 298.408(1) Å^3^.

**Figure 3:**
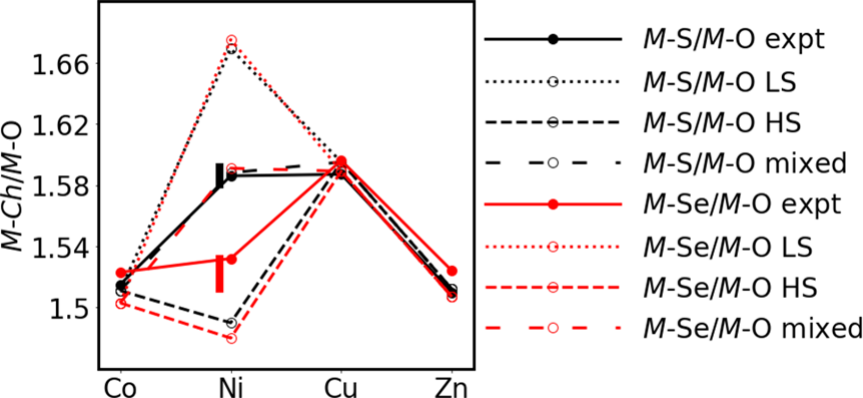
Calculated (open symbols/dashed lines) and experimental values (closed symbols/solid lines) of the *M*–*Ch*/*M*–O ratios for Sr_2_*M*O_2_Cu_2_*Ch*_2_ (*M* = Co, Ni, Cu, Zn; *Ch =* S (black), Se (red)) compounds. The experimental points are the ambient temperature values. The vertical bars indicate the range of values found experimentally for the *M*–*Ch*/*M*–O ratios for Sr_2_NiO_2_Cu_2_S_2_^11^ and Sr_2_NiO_2_Cu_2_Se_2_ between base and ambient temperatures.

Table 2 lists the experimental structural parameters for the isostructural series of compounds Sr_2_*M*O_2_Cu_2_Se_2_ (*M* = Co,^38^ Ni, Cu,^39^ Zn^40^). The key result is that the NiO_4_Se_2_ octahedron is elongated to a similar extent to the CoO_4_Se_2_ octahedron in Sr_2_CoO_2_Cu_2_Se_2_, suggesting that their electronic configurations only differ in the occupancies of largely nonbonding 3*d* orbitals and that their occupancies of the antibonding orbitals are similar: (*dz*^2^)^1^ (*dx*^2^ – *y*^2^)^1^ resulting in high-spin Ni^2+^. In contrast, the CuO_4_Se_2_ octahedron in Sr_2_CuO_2_Cu_2_Se_2_ is much more elongated than that in the Co and Ni analogues because of the (*dz*^2^)^2^ (*dx*^2^ – *y*^2^)^1^ configuration. The analogous comparison for the oxide sulfide series is tabulated in Table S1, and the experimental ratios of the axial *M–Ch* to the equatorial *M*–O distances for the selenide and sulfide series are shown graphically in Figure 3. The comparison between Sr_2_NiO_2_Cu_2_Se_2_ and the sulfide analogue is given in Table 3. There is a significant difference in the axial elongation of the NiO_4_*Ch*_2_ octahedra: the Ni–S/Ni–O ratio in Sr_2_NiO_2_Cu_2_S_2_ is much larger than the Ni–Se/Ni–O ratio in Sr_2_NiO_2_Cu_2_Se_2_. Indeed, as Figure 3 shows, this ratio in the sulfide and selenide appear consistent with Sr_2_NiO_2_Cu_2_Se_2_ containing a high-spin Ni^2+^ cation (i.e., (*dz*^2^)^1^ (*dx*^2^ – *y*^2^)^1^) and Sr_2_NiO_2_Cu_2_S_2_ containing a low-spin Ni^2+^ cation (i.e., (*dz*^2^)^2^ (*dx*^2^ – *y*^2^)^0^), which is consistent with the lack of the ordered moment and the calculations previously reported for the sulfide.^12^

**Table 2:** Comparison of Structural Parameters for Sr_2_*M*O_2_Cu_2_Se_2_ (*M* = Co, Ni, Cu, Zn)

compound	Sr_2_CoO_2_Cu_2_Se_2_	Sr_2_NiO_2_Cu_2_Se_2_	Sr_2_CuO_2_Cu_2_Se_2_	Sr_2_ZnO_2_Cu_2_Se_2_
reference	ref.^38^	this work	ref.^39^	ref.^40^
radiation	PXRD	PXRD/PND	PND	PND
*a* (Å)	4.04876(2)	4.026470(8)	3.97117(4)	4.06521(4)
*c* (Å)	18.3571(2)	18.40612(4)	18.8199(2)	18.3753(3)
*c/a*	4.53401(5)	4.57128(1)	4.73913(8)	4.52013(8)
volume (Å^3^)	300.918(4)	298.408(1)	296.792(7)	303.668(8)
*M*–O (Å) [4]^a^	2.02438(1)	2.013235(4)	1.98558(2)	2.03260(2)
*M*–Se (Å) [2]^a^	3.0827(12)	3.0843(3)	3.1698(4)	3.0968(4)
*M*–Se/*M*–O	1.5228(6)	1.5320(1)	1.5964(2)	1.5236(2)
Cu–Se (Å) [4]^a^	2.5235(7)	2.5209(2)	2.5098(2)	2.5244(3)
Se–Cu*–*Se, α (°) [2]^a^	106.69(5)	105.994(13)	104.582(15)	107.256(14)
Se*–*Cu*–*Se, β (°) [4]^a^	110.88(3)	111.237(6)	111.970(8)	110.590(7)

a The numbers in square brackets indicate the number of bonds or angles of a particular type. See Figure 1 for the definition of angles.

**Table 3:** Comparison of Lattice Parameters and Selected Bond Lengths of Sr_2_NiO_2_Cu_2_S_2_ and Sr_2_NiO_2_Cu_2_Se_2_ Compared with Sr_2_NiO_2_Cl_2_

compound	Sr_2_NiO_2_Cl_2_	Sr_2_NiO_2_Cu_2_S_2_	Sr_2_NiO_2_Cu_2_Se_2_
reference	ref (42)	ref (11)	this work
radiation	PXRD	PND	PXRD/PND
*a* (Å)	4.03896(4)	3.92159(2)	4.026470(8)
*c* (Å)	15.09293(16)	18.11558(15)	18.40612(4)
*c/a*	3.73684(5)	4.61945(4)	4.57128(1)
volume (Å^3^)	246.214(4)	278.597(5)	298.408(1)
Ni–O (Å) [4]^b^	2.01948(3)	1.96080(1)	2.013235(4)
Ni–*X*^a^ (Å) [2]^b^	2.716(3)	3.1054(8)	3.0843(3)
Ni–*X*^a^/Ni–O	1.345(1)	1.5837(4)	1.5320(1)
Cu–*X*^a^ (Å) [4]^b^		2.4230(5)	2.5209(2)
*X*^a^–Cu*–X*^a^, α (°) [2]^b^		108.04(3)	105.994(13)
*X*^a^*–*Cu*–X*^a^, β (°) [4]^b^		110.191(15)	111.237(6)

a *X* = Cl, S, and Se.

b The numbers in square brackets indicate the number of bonds or angles of a particular type. See Figure 1 for the definition of angles.

We briefly comment here on the analogous compound Sr_2_NiO_2_Cl_2_, which, apart from the absence of coinage metal in the halide layers, has a similar arrangement of ions. The absence of coinage metal leads to a large contraction along the *c*-axis in this compound, which has a much smaller unit cell than both Sr_2_NiO_2_Cu_2_Se_2_ and Sr_2_NiO_2_Cu_2_S_2_. Despite this, it has similar properties to that of the selenide, Sr_2_NiO_2_Cu_2_Se_2_. The *a* lattice parameter, which reflects the Ni–O bond lengths (*a*/2), is very similar. The contraction along the stacking direction, *c,* leads to a less-elongated Ni^2+^ octahedron in Sr_2_NiO_2_Cl_2_ than that in both the copper-containing chalcogenide analogues, which based on the arguments presented here should lead to it being high-spin. This is supported by the magnetic susceptibility which shows a distinctive feature at 210 K, suggestive of long-range magnetic order, that is, high-spin Ni^2+^. This state is predicted computationally,^41^ although such ordering has not been probed experimentally using PND to the best of our knowledge.

### Computation of Crystal and Electronic Structures

In order to gain more insight into these systems, we have performed an extended series of calculations using DFT (VASP, PBE functional – full details are given in the Experimental Section). Both lattice parameters and atomic positions were optimized for a tetragonal unit cell, where a Hubbard *U* value of 2.0 eV was applied to all open-shell transition metal ions (Co, Ni, and Cu). The lattice parameters, *M*–O and *M*–*Ch* bonds, *M*–*Ch*/*M*–O ratio, and the magnetic moment on the transition metal ion are collected in Table 4 for *M* = Ni and Table S2 for *M* = Co/Ni/Cu/Zn combinations with S and Se. The computed ratio of elongation, *M*–*Ch*/*M*–O, is also plotted in Figure 3, alongside the experimental data that are discussed above.

**Table 4:** Comparison of Experimental and Calculated Lattice Parameters and Selected Bond Lengths of Sr_2_NiO_2_Cu_2_S_2_ and Sr_2_NiO_2_Cu_2_Se_2_

	Sr_2_NiO_2_Cu_2_S_2_				Sr_2_NiO_2_Cu_2_Se_2_		
		DFT				DFT	
	exp	HS	LS	mixed	exp	HS	LS
*a*/Å^a^	5.544	5.615	5.417	5.508	5.694	5.710	5.504
*c*/Å	18.120	17.271	18.454	17.910	18.406	17.844	19.249
Ni–O/Å	1.96	1.985	1.915	1.903	2.013	2.019	1.946
				1.992			
Ni–*Ch*/Å	3.11	2.967	3.195	3.140	3.084	2.989	3.270
				3.044			
Ni–*Ch*/Ni–O	1.586	1.494	1.668	1.588	1.532	1.481	1.681
μ(Ni)	0.0	1.60	0.0	1.60	1.31(2)	1.60	0.0
				0.0			
energy/eV		+0.27	0.0	+ 0.03		0.0	+0.26

a Note that a √2*a* × √2*a* × *c* expansion of the structural cell is used for the computation.

In all cases where the transition metal is one of Co, Cu, or Zn, the correspondence between the computed structural parameters and the experimental structural data is extremely good. The most striking discrepancy is in the calculated lattice parameter *c*, which is systematically underestimated by 0.15–0.25 Å, regardless of whether the anion is S^2–^ or Se^2–^. The fact that the error is consistent across a range of transition metals and both chalcogens suggests that its origins may lie in the pseudopotential of Sr^2+^, the only common factor in all six calculations. The lattice parameter *a* is also underestimated but by a much smaller amount – typically ≈ 0.03 Å for the sulfides and somewhat less for the selenides. The result is that the *M*–*Ch*/*M*–O elongation ratio is very close to the experimental values for all six combinations involving Co, Cu, and Zn.

**Figure 4:**
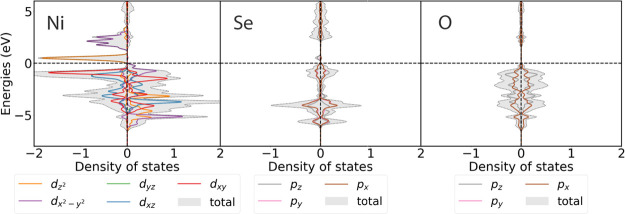
Spin-polarized densities of states for the antiferromagnetic (AFM) ground state of Sr_2_NiO_2_Cu_2_Se_2_ using a √2*a* × √2*a* × *c* expansion of the unit cell.

Matters are somewhat more complicated for Ni, where we have been able to locate two quite distinct states of similar energy, one where the Ni^2+^ ions are in local high-spin configurations (μ/μ_B_ = 1.6) and the other where they are in low-spin configurations. In the high-spin case, the local moments are coupled antiferromagnetically in a checkerboard arrangement. Convergence on these two states was controlled by the imposition of different initial spin densities using the MAGMOM keyword in VASP (values were ±2.0 for the high-spin case and 0 for low spin, although the result is not strongly dependent on the exact value used in the former). For both Ni systems (S and Se), we report the properties of both high-spin (HS) and low-spin (LS) configurations, as shown in Table 4. For the selenide, the high-spin configuration is the more stable of the two, lying 0.26 eV (per unit cell) lower in energy than the corresponding low-spin state, and the corresponding DOS is shown in Figure 4. The high-spin configuration also offers the best match to the measured structural parameters (the dashed line in Figure 3), particularly in the *ab*-plane where the Ni–O bonds are within 0.01 Å of the experimental values. In contrast to the success in replicating *a*, the computed value of *c* is underestimated by 0.55 Å, a far greater deviation from the experiment than that was observed in any of the Co/Cu/Zn systems where the error was consistently of the order of 0.25 Å. As a result, the Ni–Se/Ni–O ratio is also underestimated, the computed value being 1.481 vs a measured value of 1.536. The alternative low-spin configuration (dotted line in Figure 3), in addition to being higher in energy, also affords a much worse match with the experiment: the lattice is compressed in the *ab*-plane, and the computed *c* parameter is 0.85 Å greater than the experimental value (note that the value of *c* is always underestimated, not overestimated, in the Co/Cu/Zn systems, so such a large deviation appears entirely out of line with the general behavior of this class of compounds).

Turning to the sulfide system, the situation is even more complex. We again locate both high- and low-spin configurations, and this time, it is the latter that is more stable, lying 0.27 eV below the high-spin alternative for *U*_Ni_ = 2 eV. However, the high-spin/low-spin separation is well known to be sensitive to the chosen value of *U*, and, indeed, if we repeat the calculations with *U*_Ni_ = 4 eV, we find that the energies are reversed and that the high-spin configuration is more stable by 0.87 eV. Given this uncertainty, we do not place too much emphasis on the computed energies other than to note that for a given value of *U*_Ni_ (2.0 eV in Table 4), the low-spin configuration is relatively stabilized in the sulfide compared to the selenide, consistent with the absence of additional Bragg peaks in Sr_2_NiO_2_Cu_2_S_2_.^11^ The computed lattice parameters also present a rather ambiguous picture, with neither high-spin (dashed line) or low-spin (dotted line) forms offering convincing agreement with the experiment (see Figure 3). All the experimentally measured bond lengths and lattice parameters, along with the Ni–S/Ni–O elongation ratio, sit almost exactly between the limiting values of the high- and low-spin forms. In absolute terms, the high-spin value for *c* in Sr_2_NiO_2_Cu_2_S_2_ is underestimated by 0.85 Å, even more than that in the selenide case. The low-spin value for *c* in Sr_2_NiO_2_Cu_2_S_2_ is closer to that in the experiment (0.33 Å higher), but note again that this value is consistently underestimated, not overestimated, by the calculations for Co/Cu/Zn, where the spin state is unambiguous. If we correct the Ni–S values for this 0.25 Å underestimation, we see again that the experimental value of *c* lies almost exactly midway between the computed high- and low-spin values. This observation prompted us to perform an additional series of calculations, searching for locally stable configurations with mixed high-spin and low-spin configurations. The “mixed” entry in Table 4 and Figure 3 corresponds to one ordered example, where high-spin and low-spin Ni^2+^ ions are arranged within each layer in a checkerboard arrangement. This was achieved by first imposing different values of *U*_Ni_ (2 eV for low-spin Ni^2+^, 4 eV for high-spin Ni^2+^) and then using the converged geometry and density to initialize a calculation where *U*_Ni_ = 2 eV was imposed for all metal centers. This mixed configuration offers remarkably good agreement with experiment – *c* is now underestimated by 0.21 Å and *a* by 0.03 Å, precisely replicating the behavior of the Co/Cu/Zn systems. The Ni–O and the Ni–S distances now differ, with the high-spin (Ni–O 1.99 Å, Ni–S 3.04 Å) and low-spin values (Ni–O 1.90 Å, Ni–S 3.14 Å) values close to those computed for the all-high or all-low configurations, respectively. The Ni–S/Ni–O ratio computed using the average of the two Ni–O and the two Ni–S distances is in remarkable agreement with the experimental value (widely spaced dashed line shown in Figure 3). It is, of course, possible that other, less-ordered, arrangements of high-spin/low-spin Ni^2+^ ions would afford similar structural parameters, or indeed that the agreement between experiment and theory for the mixed state is coincidental – as we discuss below, further experimental work is required to probe these compounds in greater detail. Further calculated results for different values of *U* can be seen in Figures S5 and S6 as well as in Tables S3 and S4.

### Antiferromagnetic Ordering in Sr_2_NiO_2_Cu_2_Se_2_

Variable temperature PND at low temperatures on Sr_2_NiO_2_Cu_2_Se_2_ revealed additional reflections that were not present at RT, which were concentrated in the high *d*-spacing region, suggesting that they were magnetic in origin. These reflections were absent at 160 K and above (See Figure S7). These measurements contrast with the lack of any magnetic Bragg reflections reported^11^ for Sr_2_NiO_2_Cu_2_S_2_ even at 2 K and are consistent with the selenide having Ni^2+^ in a high-spin configuration (indeed, they confirm it) and the sulfide apparently having Ni^2+^ in a low-spin configuration.

**Figure 5:**
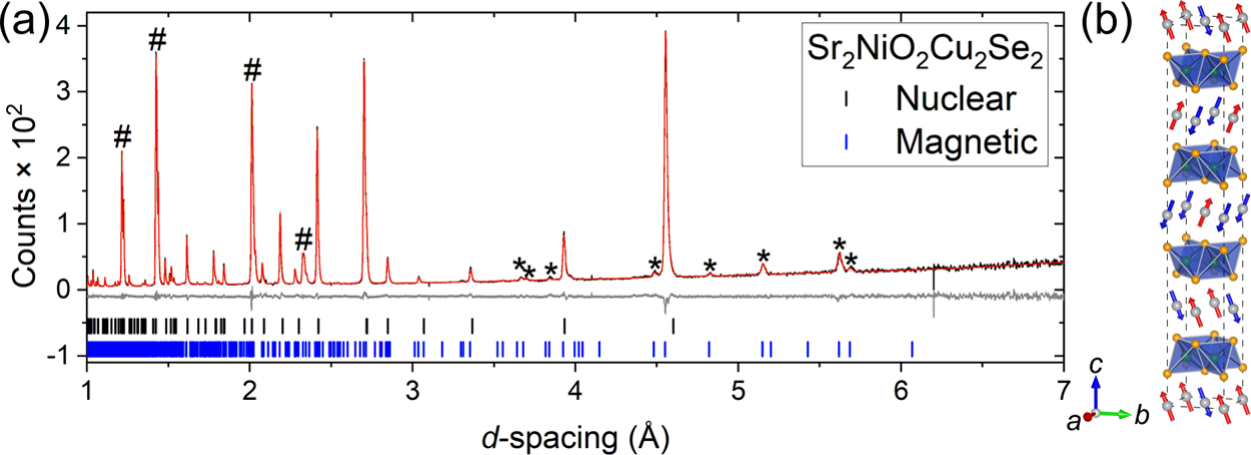
(a) Rietveld plot of the powder neutron diffraction pattern of Sr_2_NiO_2_Cu_2_Se_2_ collected using OSIRIS at 5 K. Magnetic Bragg peaks (*) and those from the Al-tailed cryostat (#) are indicated. *R*_wp_ = 7.997%; χ^2^ = 1.742 (b) Magnetic model with blue moments pointing downward and red moments pointing upward.

**Figure 6:**
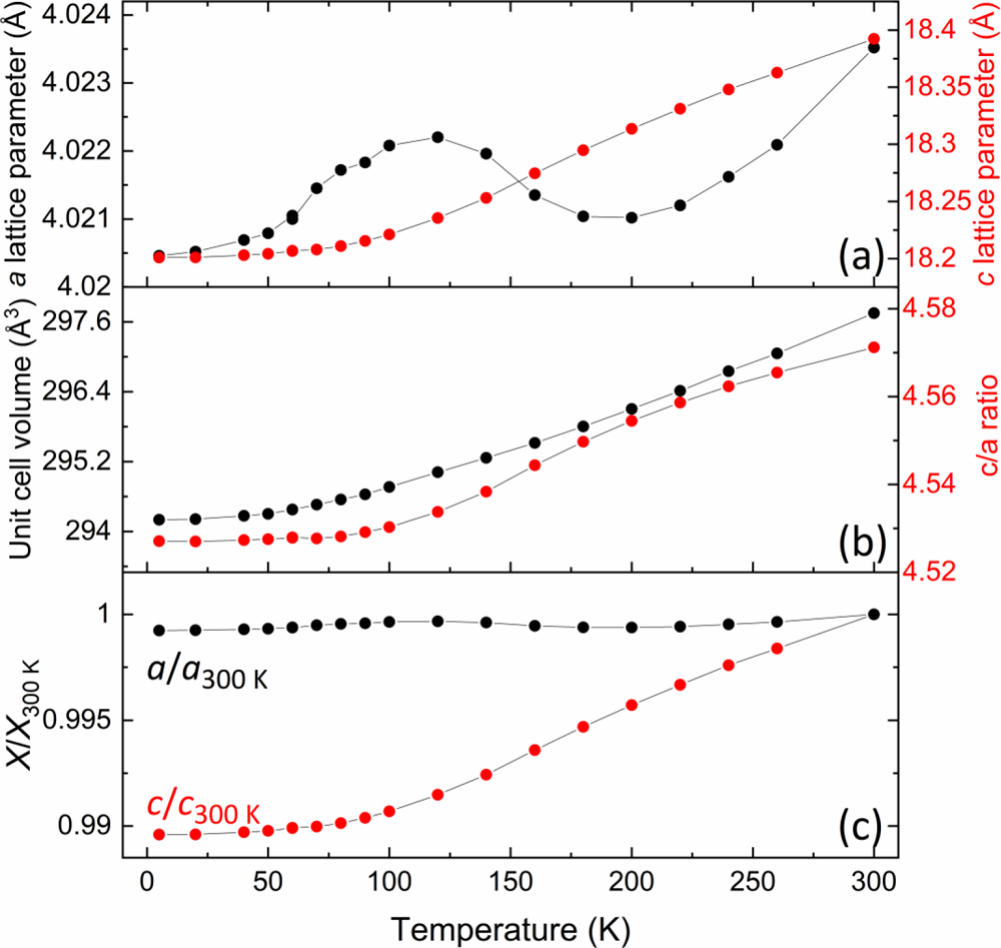
(a) Refined lattice parameters of *a* and *c* for Sr_2_NiO_2_Cu_2_Se_2_ (quenched from 850 °C) from HRPD data, (b) unit cell volume and *c*/*a* ratio, (c) *a* and *c* values normalized to their values at 300 K (bottom) to show the different relative changes in *a* and *c*. Error bars are within the plotted points.

**Figure 7:**
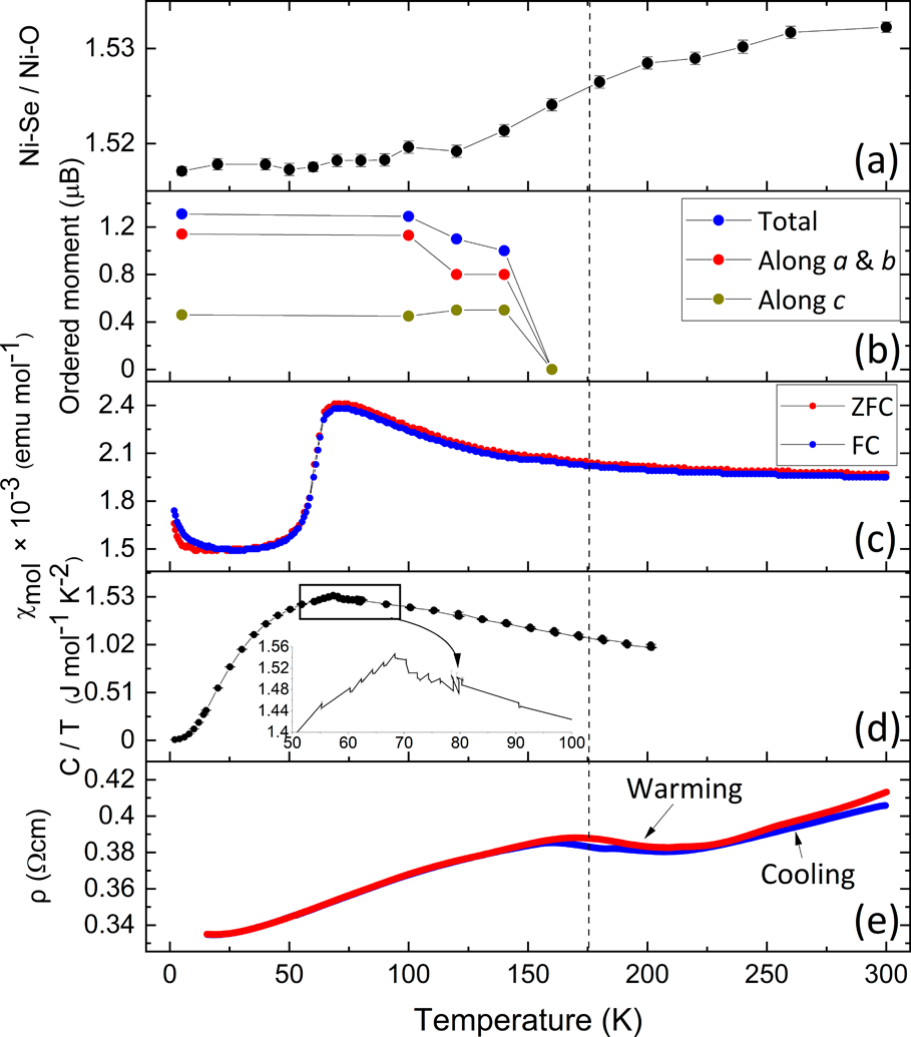
(a) Decrease in the axial elongation of the NiO_4_Se_2_ octahedron on cooling. (b) Development of long-range magnetic order on the Ni sublattice. (c) Magnetic susceptibility of Sr_2_NiO_2_Cu_2_Se_2_ (quenched from 950 °C and containing no ferromagnetic impurity) showing a broad feature around 150–170 K (clearer in the derivative plot in Figure S11) and a sharp drop at around 70 K. (d) Heat capacity of Sr_2_NiO_2_Cu_2_Se_2_ measured in zero applied field with the inset showing a clear feature coincident with the sharp drop in susceptibility. (e) Bulk resistivity of Sr_2_NiO_2_Cu_2_Se_2_ (quenched from 850 °C), measured in a constant current of 10 mA as a function of temperature (15–300 K), showing a transition around the temperature of long-range magnetic ordering. The dashed line is a guide to this transition.

All the magnetic peaks of Sr_2_NiO_2_Cu_2_Se_2_ could be indexed using a √2*a* × √2*a* × 2*c* expansion of the nuclear unit cell. Starting from magnetic space group *P*1 (1.1), the activation of possible magnetic ordering modes was tested in ISODISTORT.^27^ Activation of the mP4(a) and mX3^+^(a) modes (Figure S8–10) was adequate for modeling the data, leading to magnetic space group *P_C_*4_1_2_1_2 (92.117 in the Belov–Neronova–Smirnova (BNS) scheme).^43,44^ The Rietveld refinement at 5 K is shown in Figure 5a, with the model shown in Figure 5b. The refined Ni^2+^ moment had a magnitude of 1.31(2) μ_B_ at 5 K (μ_*a*_ = μ_*b*_ 0.46 *μ*_B_; μ_*c*_ = 1.14(2) μ_B_; Table S5)), smaller than the 2 μ_B_ expected for high spin Ni^2+^, and this reduction is likely due to the effects of covalency. The value is comparable to the calculated spin density of 1.60 μ_B_ per Ni^2+^ obtained for the antiferromagnetic state from the calculations, confirming that Ni^2+^ is in the high-spin state in Sr_2_NiO_2_Cu_2_Se_2_. The thermal evolution of structural parameters is shown in Figure 6 and thermal evolution of the refined moment is shown in Figure 7b.

### Changes in the Crystal Structure of Sr_2_NiO_2_Cu_2_Se_2_ with Temperature

The lattice parameters of Sr_2_NiO_2_Cu_2_Se_2_ as a function of temperature were extracted from Rietveld refinement against PND data (HRPD), and the values are plotted in Figure 6. The *c* lattice parameter decreases on cooling by significantly more (1.04%) than the *a* lattice parameter (0.08%). The shapes of the unit cell volume and *c*/*a* curves are dominated by the change in *c* because *a* remains relatively static across the full temperature range; however, as shown in Figure 6, it does not vary in a smooth fashion. The Ni coordination environment (Figures 3 and 7a) becomes less elongated on cooling to an extent that is much greater than what would be expected from thermal contraction effects. Between 250 K and about 120 K, the change is particularly rapid, explaining the faster-than-expected contraction in the *c* lattice parameter as well as the expansion in the *a* lattice parameter in this regime. This temperature regime is close to the magnetic ordering temperature, suggesting a relationship between the onset of magnetic ordering and the coordination environment of the Ni ion becoming less elongated. The 1% decrease in the Ni–Se/Ni–O ratio on cooling from 300 to 5 K is much larger than those for isostructural Co systems (0.40% in Sr_2_CoO_2_Cu_2_Se_2_ and 0.55% in Sr_2_CoO_2_Cu_2_S_2_).^45^ The base temperature value for the Ni–Se/Ni–O ratio from the experimental data matches the calculated value closely, and for all the transition metals from Co to Zn, there is very close agreement between the experiment and calculation for the selenide systems (see Figure 3), as discussed above. In contrast, the Ni–S/Ni–O ratio in Sr_2_NiO_2_Cu_2_S_2_ undergoes a sharp increase on cooling,^45^ although the range of values for the Ni–S/Ni–O ratio as a function of temperature is much smaller than the large difference between the experimental and calculated values in this case, as shown by the bar in Figure 3.

### Thermal Evolution of Physical Properties of Sr_2_NiO_2_Cu_2_Se_2_

#### Magnetometry

The magnetic susceptibility of Sr_2_NiO_2_Cu_2_Se_2_ (quenched from 850 °C) was measured using a 40–30 kOe subtraction (as described in the Experimental Section) in order to minimize the effects of a small ferromagnetic impurity, likely to be a minuscule amount of elemental Ni. A purer sample (quenched from 950 °C) was also measured in a smaller field (10 kOe) and contained no measurable ferromagnetic impurity, so the subtraction was not required. The samples showed very similar behavior. The magnetic susceptibility curve, shown in Figure 7c, reveals two main features. The most obvious feature is the sharp drop in magnetic susceptibility on cooling at approximately 70 K (Figure S11), which accompanies the final shortening of the *a* parameter on cooling. There is also a less distinct broad feature at 150–170 K (more visible in Figure S11). This coincides with the appearance of magnetic Bragg peaks in the PND data (Figures 7b and S7), and so we identify this feature as coinciding with the Néel temperature (*T*_N_). Consistent with the fairly high value *T*_N_ compared with the measuring range, the inverse susceptibility is nonlinear, even in the region around room temperature, so Curie or Weiss constants cannot be extracted.

The magnetic susceptibility of Sr_2_NiO_2_Cu_2_Se_2_ is also compared with that of its sulfide analogue, Sr_2_NiO_2_Cu_2_S_2_^11^ (measured under similar conditions), as shown in Figure S12. The susceptibility of Sr_2_NiO_2_Cu_2_S_2_ drops sharply at around 160 K (a feature superficially resembling that at ≈ 70 K in the selenide), and then exhibits a sharp rise at about 130 K which is not mirrored in the selenide. Given the different spin states for the two compounds, it is not clear that there is any meaningful correspondence between the susceptibilities of the two compounds, but we discuss this further below. There is no indication from the literature that the drop in susceptibility is due to the small amount of Cu_0.8_Ni_0.2_ impurity. Copper–nickel alloys have been well studied, and it has been shown that there is in fact a discernible upturn in the susceptibility at low temperatures when the percentage of Ni surpasses ≈ 10%.^46^

#### Resistivity

The resistivity of Sr_2_NiO_2_Cu_2_Se_2_ (Figure 7e) decreases on cooling, consistent with metallic behavior. A hump, with hysteresis dependent on whether the sample was being measured on cooling or heating, is apparent in the temperature range 160–215 K. This coincides with the less distinct feature in the magnetic susceptibility curve, identified as *T*_N_. There is no feature in the resistivity that corresponds to the sharp transition in the magnetic susceptibility (≈ 70 K).

#### Heat Capacity

Heat capacity measurements revealed a feature coinciding with the sharp drop in the magnetic susceptibility at 70 K (see Figure 7d). The estimated entropy change at this transition was 0.84(3) Jmol^–1^ K^–1^ (≈ 0.1 R). There was no clear feature evident at higher temperatures corresponding to *T*_N_, although further measurements would be required to investigate whether a broad feature due to the magnetic transition is evident above the lattice background.

### Structural Response of Sr_2_NiO_2_Cu_2_Se_2_ to Applied Pressure

**Figure 8:**
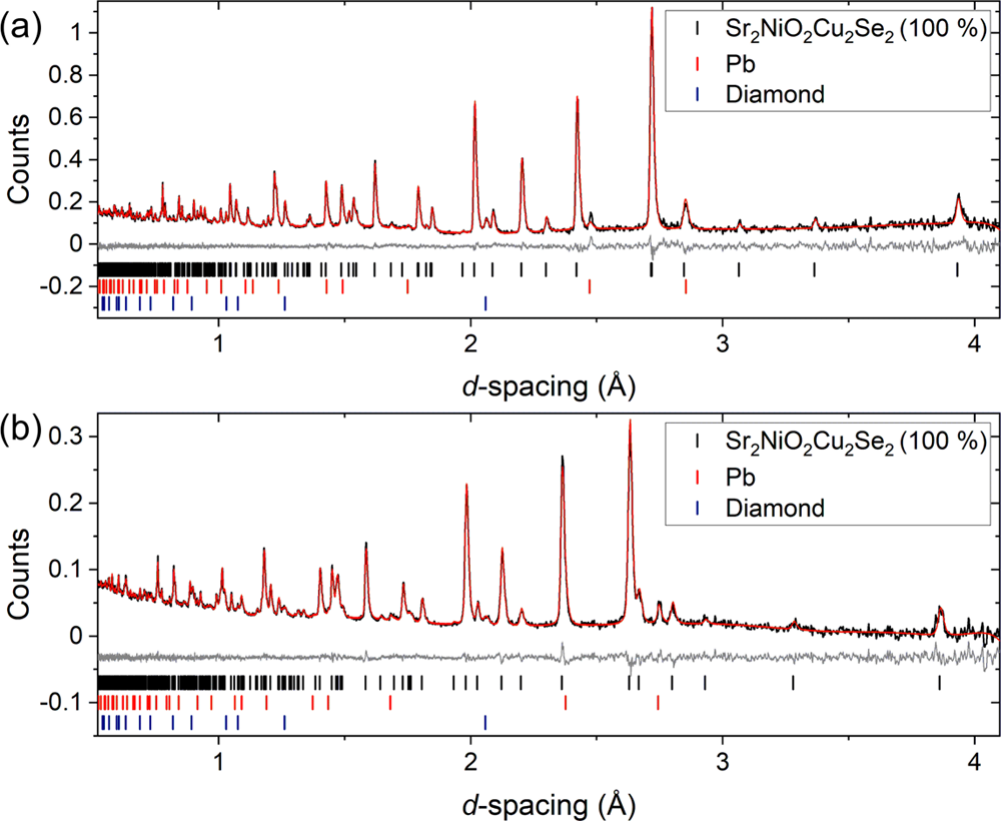
(a) PND of Sr_2_NiO_2_Cu_2_Se_2_ (quenched from 950 °C), at ambient pressure *R*_wp_ = 4.771%; χ^2^ = 0.751 (b) 7.2 GPa (obtained with an applied load of 90 tonnes in the Paris–Edinburgh cell) *R*_wp_ = 4.793%; χ^2^ = 0.744, measured on the PEARL diffractometer at ISIS. It shows the experimental data in black, the fit from the model in red, and the difference between the two in gray.

PND measurements on PEARL were used to probe changes in the crystal structure of Sr_2_NiO_2_Cu_2_Se_2_ as a function of applied pressure. In particular, we were keen to investigate whether applying pressure and increasing the ligand field strength could drive Sr_2_NiO_2_Cu_2_Se_2_ into the low-spin regime found in the sulfide analogue. Low-temperature measurements down to 100 K on PEARL were accessed by immersing the Paris–Edinburgh cell in a liquid nitrogen bath, but it was clear in performing these low-temperature measurements that the magnetic Bragg scattering in Sr_2_NiO_2_Cu_2_Se_2_ at this temperature was not sufficiently intense to be observable above the background noise because of the small sample size and the relatively small, long-range-ordered moment. Hence, monitoring changes in the magnetic scattering under the application of pressure was not possible. Two initial measurements (run A and run B) focusing on the low-pressure region were performed at ambient temperature using Zirconia-toughened alumina (ZTA) anvils. The load was increased in steps of 5 tonnes to reach an applied pressure of 2.2 GPa before being increased in smaller increments of 2 tonnes (which translated to refined pressure increases of approximately 0.1–0.2 GPa). During run B, sintered diamond (SD) anvils were also used to apply a force of 50, 60, and 70 tonnes to study the high-pressure region, which resulted in a maximum applied pressure of 7.3(1) GPa. During run A, analysis of the low-pressure region showed that there was a decrease in the rate of change of the *c*/*a* ratio (Figure S13). This behavior was also observed in the Ni–Se bond distance at an applied pressure or 3.2 and 4 GPa for run A and run B (Figure S14), respectively, which was indicative of a possible structural anomaly which warranted a further study. These anomalies led us to prepare a fresh portion of a different sample of Sr_2_NiO_2_Cu_2_Se_2_ which was then pressed at ambient temperature (run C) up to 7.2(1) GPa using only SD anvils with the refinements against the data collected at ambient pressure and at 7.2(1) GPa displayed in Figure 8a,b. Over the full range of pressures explored, there was no obvious anomaly in the behavior of the lattice parameters or the structural parameters, suggesting that up to pressures of 7.2(1) GPa, there is no change in the Ni^2+^ spin state and that the observed anomalies in the data during both runs A and B were experimental artifacts. A transition from the high-spin to low-spin regime would be accompanied by a shortening of the Ni–O distance (i.e., the *a* lattice parameter) and an increase in the Ni–Se/Ni–O ratio, as seen in the experimental and computational comparison between Sr_2_NiO_2_Cu_2_S_2_ and Sr_2_NiO_2_Cu_2_Se_2_, because of an electron moving from the *dx*^2^ – *y*^2^ to the *dz*^2^ orbital. Significant changes in the bond lengths are not observed. Evidently, the increase in the ligand field strength under a pressure of 7.2(1) GPa is not sufficient to drive the selenide system into the low-spin regime, although we cannot rule out that such a transition might occur at higher pressures.

**Figure 9:**
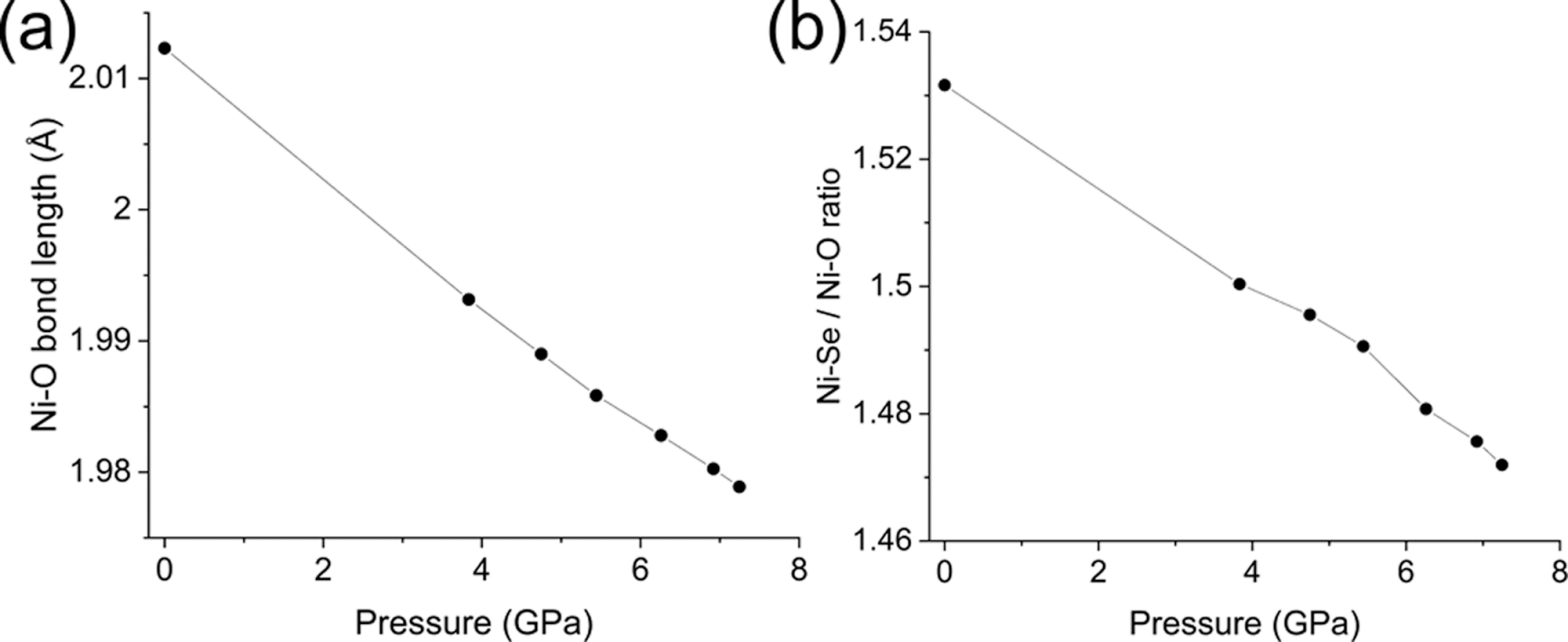
(a) Plots of the Ni–O bond distance (= *a*/2) along with (b) the Ni^2+^ environment as a function of pressure focusing on the high-pressure region. Error bars for the Ni–O bond lengths are within the data points due to the precision with which the lattice parameters can be refined.

**Figure 10:**
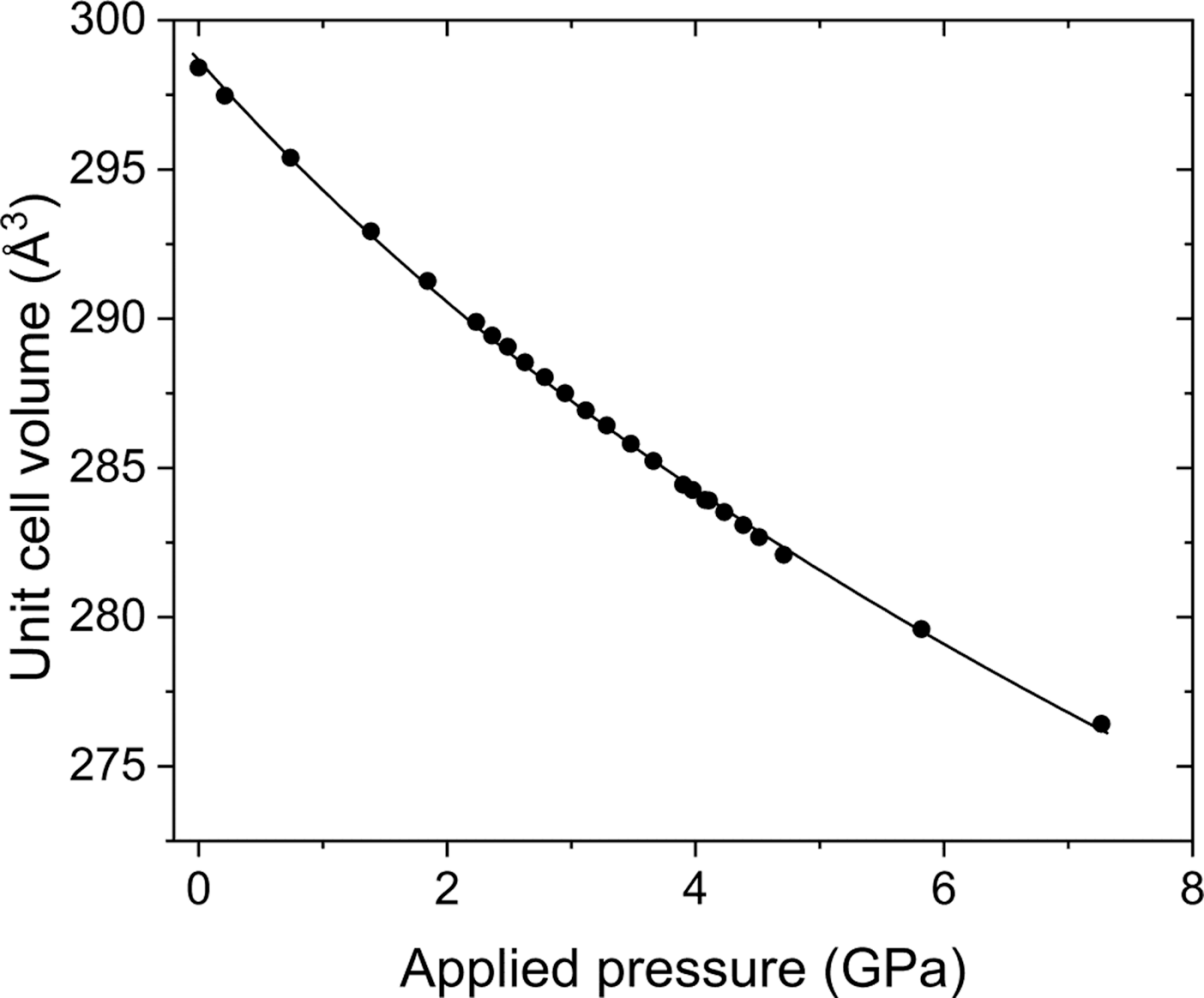
Variation with pressure of the cell volume of Sr_2_NiO_2_Cu_2_Se_2_. Error bars reside within the data points. Solid line is the fit to the data using a third-order Birch–Murnaghan EoS (eq 1).

Table 5 displays some refined parameters from the data measured on loading and at 7.2(1) GPa with the complete data set for the whole pressure sweep displayed in Figure 9 and Table S6. Further plots can be seen in the Supporting Information (Figures S15–S17); the smooth variation of the volume, *V*, as a function of pressure, *P*, across the series indicated that the dependence could be fitted to a third-order Birch–Murnaghan equation of state^47^ (eq 1, Figure 10).
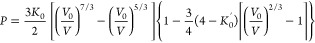
1

**Table 5:** Refined Parameters for Sr_2_NiO_2_Cu_2_Se_2_ and Pb from the Refinements in Figure 8

applied pressure/GPa	0	7.2(1)
*a* lattice parameter (Å)	4.02456(5)	3.9577(6)
*c* lattice parameter (Å)	18.3844(5)	17.5782(5)
Ni–O bond length (Å)	2.01228(3)	1.97888(3)
*c*/*a*	4.5681(1)	4.4415(2)
Pb volume (Å)^3^	120.76(7)	107.30(5)

Using the EosFit7-GUI software,^48^ the following parameters were obtained: *V*_0_ = 298.66(15) Å^3^, the bulk modulus *K*_0_ = −*V*_0_(∂*P*/∂*V*) = 63.1(18) GPa, and its first pressure derivative *K*_0_*′* = 10.5(8) (Figures 10 and S18). Similar treatment of the cell parameters *a* and *c* can also be analyzed by treating them as a volume component, that is, *a*^3^ and *c*^3^, which resulted in two directional compressibilities. The *a*-axis yields a bulk modulus of *K*_0_(*a*) of 119(3) GPa and a *K*_0_*′*(*a*) of 1.7(13), whereas the *c*-axis has a bulk modulus *K*_0_(*c*) of 42.8(6) GPa with a *K*_0_*′*(*c*) = 3.2(3) (see supporting Figures S19 and S20).

The *ab*-plane being less compressible than the *c*-axis is a common feature of layered structures, whereby there tends to be higher atom density as well as bonds lying parallel to the *ab*-plane such as the Ni–O bonds in the compounds described in this study, which greatly restricts the compression along this direction. These differing compressibilities led to a smooth reduction in the *c*/*a* ratio of 2.77% as the pressure is increased to 7.2 GPa (see Supporting Information Figure S16).

### Evolution of the Crystal Structure and Magnetic Ordering across the Series Sr_2_NiO_2_Cu_2_(Se_1–*x*_S_*x*_)_2_

**Figure 11:**
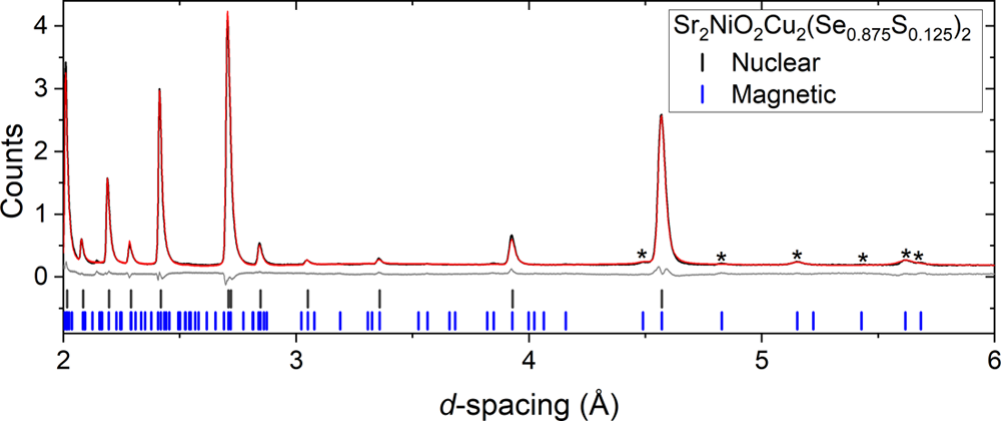
Rietveld refinement at 1.5 K of Sr_2_NiO_2_Cu_2_(Se_0.875_S_0.125_)_2_ against bank 3/8 of the WISH diffractometer at ISIS. *R*_wp_ = 4.544%; χ^2^ = 0.020.

**Figure 12:**
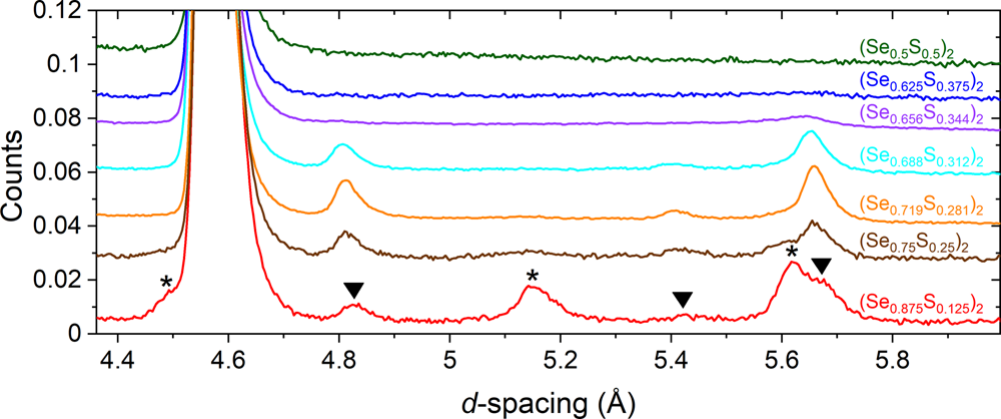
Normalized raw data for Sr_2_NiO_2_Cu_2_(Se_1–*x*_S_*x*_)_2_ (0.125 ≤ *x* ≤ 0.5) from bank 3/8 of the WISH diffractometer at ISIS.

**Figure 13:**
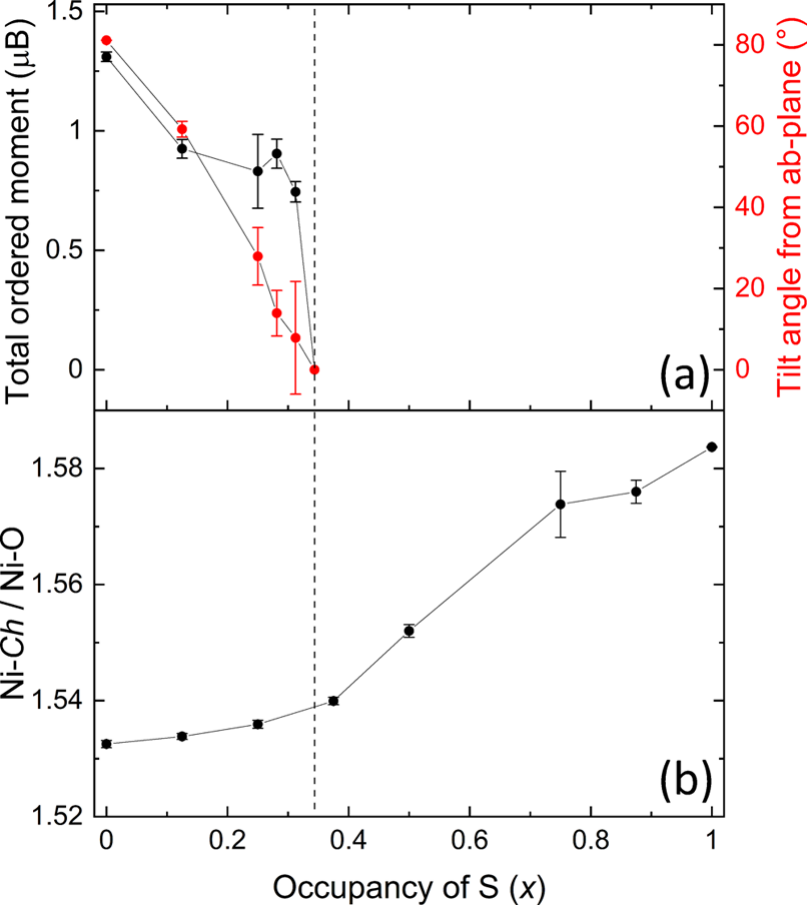
(a) Change in the magnitude of the total ordered moment and the tilt angle from the *ab-*plane along with (b) the Ni–*Ch*/Ni–O ratio as a function of Se occupancy in Sr_2_NiO_2_Cu_2_(Se_1–*x*_S_*x*_)_2_. Dashed line is a guide to the eye, whereby the loss of the long-range order coincides with an upturn in the axial elongation of the Ni^2+^ environment.

The fact that there are magnetic Bragg peaks observed for Sr_2_NiO_2_Cu_2_Se_2_, whereas there were none observed for Sr_2_NiO_2_Cu_2_S_2_ at low temperatures, together with the very different Ni–*Ch*/Ni–O distance ratios in the two compounds, suggested that in the series Sr_2_NiO_2_Cu_2_(Se_1–*x*_S_*x*_)_2_, a high-spin to low-spin transition would be evident as a function of composition with increasing *x* by monitoring the structural parameters and/or the magnetic behavior (Figures S21–S25 and Tables S7–S11). One gram of four initial samples, Sr_2_NiO_2_Cu_2_(Se_0.875_S_0.125_)_2_, Sr_2_NiO_2_Cu_2_(Se_0.75_S_0.25_)_2_, Sr_2_NiO_2_Cu_2_(Se_0.625_S_0.375_)_2_, and Sr_2_NiO_2_Cu_2_(Se_0.5_S_0.5_)_2_, were measured on the WISH diffractometer at ISIS.^25^ The high signal-to-noise ratio at long *d-*spacings achievable on the instrument meant that even the relatively weak magnetic peaks found in these compounds are observable. A representative Rietveld refinement is shown in Figure 11 with further refinements shown in the Supporting Information (Figures S26–S28). It was clear that for the *x* = 0.375 sample and more S-rich compositions that there was no magnetic scattering measurable above the noise level, but there was significant evolution of the magnetic peaks in the range 0 ≤ *x* ≤ 0.25 before their disappearance by *x* = 0.375, which was suggestive of a change in the magnetic structure. Therefore, to pinpoint the changes and the transition to the state with no observable magnetic long-range order with greater accuracy, samples with higher compositional resolution were probed. Three further samples Sr_2_NiO_2_Cu_2_(Se_0.719_S_0.281_)_2_, Sr_2_NiO_2_Cu_2_(Se_0.688_S_0.312_)_2_ and Sr_2_NiO_2_Cu_2_(Se_0.656_S_0.344_)_2_ were measured on WISH, with measurements made from 1.5 K up to the Néel temperature. The normalized raw data for all seven samples at 1.5 K are shown in Figure 12. On inspection of the magnetic Bragg peaks, it was apparent that they could be grouped into two different sets, similar to those in the end member Sr_2_NiO_2_Cu_2_Se_2_ (i.e., *x* = 0) (see Figure S8). The peaks marked with an asterisk in Figure 12 decrease in intensity with increasing S content, whereas the peaks marked with an inverted triangle initially increased in intensity and then subsequently decreased. The magnetic model for Sr_2_NiO_2_Cu_2_Se_2_ was used as the starting point for the refinements of the solid solution; this model has Ni^2+^ moments that are modeled on a √2*a ×* √2*a ×* 2*c* expansion of the nuclear cell and activating the mX3+ mode (to give intensity to peaks marked with inverted triangles in Figure 12) and mP4 mode (asterisks in Figure 12), accounted for all the peaks in the diffractogram. From Figure 12, it can then be inferred from the changes in intensity that upon increasing *x* in Sr_2_NiO_2_Cu_2_(Se_1–*x*_S_*x*_)_2_, the Ni^2+^ moments undergo a spin reorientation toward the basal plane as the magnitude of the mP4 mode decreases, and this is quantified by the Rietveld refinements (Figure 13a). There is no evidence for a spin reorientation for any composition as a function of temperature. The magnetic moment is proportional to the square root of the intensity, and as can be seen from Figure 12, there is no great discernible reduction in the total integrated intensity before the magnetic peaks disappear, and so the refined values of the magnitude of the ordered moment in Figure 13a are relatively constant.

**Figure 14:**
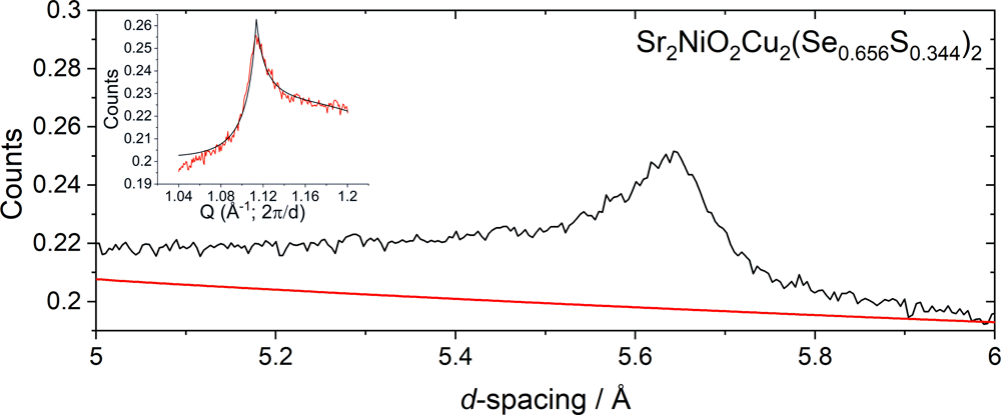
Zoomed-in region of the Rietveld refinement of Sr_2_NiO_2_Cu_2_(Se_0.65625_S_0.34375_)_2_ from bank 2/9 of the WISH diffractometer at ISIS showing additional intensity not accounted for by the nuclear model (red). Inset shows this additional peak fitted to a Warren-like peak function.

There were no magnetic Bragg peaks observed for Sr_2_NiO_2_Cu_2_(Se_0.656_S_0.344_)_2_; however, there was additional intensity around the same *d*-spacing as in the main magnetic reflection in the more Se-rich phases in the form of a highly asymmetric peak which disappeared on warming (see supporting Figure S29). The integrated intensity of this feature at 1.5 K was much weaker than that in the more Se-rich phases. It was indexed as the 100 reflection on a √2*a ×* √2*a × c* expansion of the nuclear cell. This peak profile has been seen in related compounds such as Sr_2_MnO_2_Cu_1.8_Te_2_^49^ as well as Sr_2_MnO_2_Mn_2_As_2_.^50^ It was fitted using a Warren-like function (Figure 14 inset and Figure S30) in *Q-*space. It shows a good fit to the peak at both high and low *Q*. This is consistent with antiferromagnetic nearest-neighbor ordering of high-spin Ni^2+^ ions with an estimated correlation length of 100(5) Å. We infer that there is an increasing number of low-spin diamagnetic Ni^2+^ ions present in the compound as the sulfide content increases, and so antiferromagnetic long-range order is disrupted by the Ni^2+^ spin-state disorder, hence the Warren-type peak and the reduced intensity of the magnetic scattering. For more S-rich compounds, there is no additional intensity observed compared with the nuclear model, and so we conclude that close to Sr_2_NiO_2_Cu_2_(Se_0.656_S_0.344_)_2_, there is a change in the bulk magnetic behavior of the compounds consistent with the Ni^2+^ ions becoming low spin, and this is consistent with the sharp increase in the Ni–*Ch*/Ni–O ratio as *x* increases (Figure 13b) even as the amount of the smaller chalcogenide increases.

## Discussion and Conclusions

Sr_2_NiO_2_Cu_2_Se_2_ contains Ni^2+^ ions with localized moments in planar NiO_2_ sheets. The Ni^2+^ moments in Sr_2_NiO_2_Cu_2_Se_2_ are in a highly elongated NiO_4_Se_2_ octahedron and order antiferromagnetically below 160 K. The behavior of this compound is similar to that of the isostructural Ba_2_NiO_2_Ag_2_Se_2_.^12^ The observations suggest that the Ni^2+^ ion is in a high-spin state and there was no structural signature of a potential change in the spin state for Sr_2_NiO_2_Cu_2_Se_2_ under the application of a pressure of up to 7.2(1) GPa. In contrast, Sr_2_NiO_2_Cu_2_S_2_^11^ does not exhibit any magnetic Bragg peaks and has a more elongated NiO_4_S_2_ octahedron with Ni–O bonds that are 2.6% shorter than those in the NiO_4_Se_2_ octahedron of Sr_2_NiO_2_Cu_2_Se_2_, making *dx*^2^ – *y*^2^ more antibonding, and the Ni–S distance in Sr_2_NiO_2_Cu_2_S_2_ is slightly longer than the corresponding Ni–Se distance in Sr_2_NiO_2_Cu_2_Se_2_, making *dz*^2^ less antibonding. In the sulfide, the much more elongated octahedron is consistent with a low-spin configuration for Ni^2+^ (i.e., diamagnetic ions with *dx*^2^ – *y*^2^ unoccupied), which presents an obvious plausible reason for the lack of long-range magnetic ordering.

According to the detailed DFT calculations presented here performed using different values for *U* (which quantifies on-site electron–electron repulsions), it was found that the high-spin state ((*dz*^2^)^1^ (*dx*^2^ – *y*^2^)^1^) was indeed favored for *U* = 2 eV and for higher values for Sr_2_NiO_2_Cu_2_Se_2_. In contrast, for the sulfide Sr_2_NiO_2_Cu_2_S_2_, a comparable set of calculations showed that the low-spin state ((*d*z^2^)^2^) was marginally favored for *U* = 2 eV. The computational model used replicates the structural data extremely well for the Co, Cu, and Zn analogues (both sulfide and selenide), and so we are confident that it captures the important structural features for this class of compound. However, the calculated Ni^2+^ environments show that the selenide is in fact less elongated than it should be if it was fully high-spin, and the optimized calculated structure for the sulfide in the low-spin state has an even more elongated NiO_4_S_2_ environment than that is found experimentally. The calculations are thus fairly consistent with the inference about the spin states from the structural data and the occurrence, or not, of magnetic ordering but suggest that in both compounds the situation is finely balanced and that the simple high-spin vs low-spin binary choice for the spin state is most likely a simplification. We note that in other work, a ground state consistent with *S* = 1 (i.e., high spin) was suggested for both Ba_2_NiO_2_Ag_2_Se_2_ and Sr_2_NiO_2_Ag_2_Se_2_ but, while the former showed a magnetic long-range order, no magnetic Bragg peaks were observed even at 6 K in Sr_2_NiO_2_Ag_2_Se_2_, and so it was hypothesized to be in a spin-glass state.^12^

In view of the differences between the experimental and computational environments for Ni, particularly in Sr_2_NiO_2_Cu_2_S_2_, further computations were performed, and a model involving a 50/50 split of high- and low-spin Ni^2+^ cations in a checkerboard arrangement led to calculated results that almost replicate the experimental data for Sr_2_NiO_2_Cu_2_S_2_ along with underestimations in the experimental lattice parameters much more in line with those found for the Co, Cu, and Zn analogues. While this result could be somewhat fortuitous, it would suggest that Sr_2_NiO_2_Cu_2_S_2_ contains a mixture of high-spin and low-spin Ni^2+^ cations, and Ni^2+^ spin disorders result in a loss of magnetic Bragg peaks. Further local probe experiments such as muon-spin rotation spectroscopy or ^61^Ni Mössbauer spectroscopy may help in investigating this further.

The change in behavior between the Sr_2_NiO_2_Cu_2_Se_2_ and Sr_2_NiO_2_Cu_2_S_2_ end members was observed directly as a function of composition in the solid solution Sr_2_NiO_2_Cu_2_(Se_1–*x*_S_*x*_)_2_. PND data showed that compositions up to and including *x* = 0.344 showed magnetic scattering, and analysis of the magnetic Bragg scattering as a function of increasing sulfide content showed a smooth reorientation of the spin direction toward the *ab*-plane. In the Sr_2_NiO_2_Cu_2_(Se_0.656_S_0.344_)_2_ sample, vestigial magnetic scattering is evident as a highly asymmetric Warren peak indicative of short-range ordering. For more sulfide-rich systems with *x* = 0.375 and higher, no evidence for magnetic ordering was found. Consistent with the behavior of the magnetic ordering, there is a structural signature of the change from broadly high-spin to broadly low-spin behavior as selenide is replaced by sulfide (Figure 12b). The Ni–*Ch*/Ni–O ratio (with the mixture of sulfide and selenide ions located on a single average site) shows a very gentle upturn on increasing S content up until *x* = 0.375 (the first composition which does not have magnetic Bragg scattering) and then increases more sharply as the S content increases further. This is consistent with the Ni ions remaining at the high-spin state in the Se-rich compositions, and accordingly, in the Se-rich phases that show a long-range magnetic order, there is only a small decrease in the long-range ordered moment carried by the Ni^2+^ ions. For the more sulfide-rich compositions, the Ni–*Ch*/Ni–O ratio increases sharply, even though the mean chalcogenide ion radius decreases; thus, the Ni environment clearly becomes more axially elongated, consistent with the formation of low-spin Ni^2+^ ions. Diffraction only probes the average structure, so in the phases containing both sulfide and selenide ions, one could anticipate a mixture of high-spin Ni^2+^ ions in NiO_4_Se_2_ elongated octahedra and low-spin Ni^2+^ ions in NiO_4_S_2_ octahedra, and one or other of the spin states in *C*_4*v*_ NiO_4_SSe environments. The displacement ellipsoid for Ni is quite elongated in all cases; however, refinements using a split-site model with Ni ions in *C*_4*v*_ symmetry sites located closer to one chalcogenide ion than the other resulted in a worsening of the fit and agreement factors. This spin state disorder arising inherently from the chemical disorder associated with making a solid solution would be expected to disrupt the long-range magnetic ordering found in the selenide-rich phases, leading to a loss of magnetic Bragg peaks as the sulfide content increases, as is observed. The sample Sr_2_NiO_2_Cu_2_(Se_0.656_S_0.344_)_2_ showing the Warren peak is the most S-rich composition measured that has antiferromagnetic ordering with a significant correlation length. The sulfide end member Sr_2_NiO_2_Cu_2_S_2_ has only one type of Ni environment, and while the attractive conclusion is that the lack of the magnetic long-range order^11^ in this case is because this compound contains only diamagnetic Ni^2+^ ions, the computational results and the fact that there are low-temperature structural and magnetic anomalies mean that we cannot rule out that Sr_2_NiO_2_Cu_2_S_2_ has Ni in a spin state that is intermediate between the high and low spin states.

Indeed, both Sr_2_NiO_2_Cu_2_Se_2_ and Sr_2_NiO_2_Cu_2_S_2_ display further subtleties in the details and temperature dependence of their crystal structures and some of their properties. In Sr_2_NiO_2_Cu_2_Se_2_, the computation reproduced very well the experimental Ni–O and Ni–Se bond lengths, and the magnitude of the calculated spin density localized on Ni in the antiferromagnetic state coincided with the magnitude of the long-range ordered magnetic moment. However, examination of the changes in the Ni coordination environment as a function of temperature shows that above the region of the magnetic ordering transition, the NiO_4_Se_2_ octahedron is slightly more elongated than one would expect by analogy with isostructural compounds, and it becomes less elongated on cooling and this is reflected in the behavior of the lattice parameters. Furthermore, the compound shows a high-temperature resistivity that is characteristic of metallic behavior, and this also shows an anomaly around the magnetic ordering transition in which the resistivity increases slightly on cooling, before decreasing again on further cooling. An attractive interpretation of this is that the metallic behavior arises from hole states in the top of the selenide-based band, formed by some transfer of electrons into the energetically nearby 3*dz*^2^ band of Ni (i.e., selenide slightly reduces the Ni cations) which from Figure 4 are shown to be at similar energies above *E*_F_. This would result in a more elongated NiO_4_Se_2_ octahedron than expected (more electrons in Ni/Se antibonding states), and it is well known that compounds with similar CuSe layers can readily accommodate holes in anti-bonding states at the top of the Cu-3*d*/Se-4*p*-based band, leading to metallic behavior.^51,52^ The upturn in the resistivity and the decrease in the axial elongation of the Ni octahedron on cooling would then correspond to partial electron transfer from Ni 3*dz*^2^ to Se, or they may also be associated with the onset of the long-range antiferromagnetic order.

It is not trivial to account for the anomaly in the magnetic susceptibility at 70 K in Sr_2_NiO_2_Cu_2_Se_2_, which has associated with it a significant heat capacity anomaly and coincides with the final decrease in the Ni–O distance (i.e., *a* lattice parameter) because these anomalies occur below 100 K, where the ordered antiferromagnetic moment is already saturated, and they show no structural or resistivity signature according to our measurements. The high-resolution data from the backscattering detector of HRPD do not reveal any structural distortion nor do the magnetic scattering data suggest any spin reorientation or change in the size of the magnetic moment between 100 and 5 K. We propose that measurements on oriented single crystals would be required to investigate the 70 K anomalies and the transport properties, including their anisotropy, in greater detail.

In conclusion, the synthesis, structure, and physical properties of Sr_2_NiO_2_Cu_2_Se_2_ and the solid solution Sr_2_NiO_2_Cu_2_(Se_1–*x*_S_*x*_)_2_ are reported here, and the comparison with the sulfide end-member Sr_2_NiO_2_Cu_2_S_2_ and related compounds underpinned by detailed computational investigations show that the structural and physical properties of these layered nickel oxide chalcogenides are complex; there is competition between spin states which can be tuned by chemical substitutions to tune the ligand field. These measurements on polycrystalline samples underline the complex behavior in these systems as functions of temperature and composition resulting from the fact that not only is there a choice of accessible Ni spin states but also that the chalcogenide states are in close energetic proximity to the Ni^2+^ frontier states. The contrasting thermal evolution of the structural, magnetic, and transport properties of the sulfide and selenide end members, together with the evolution of the structure and magnetism across the solid solution and the computational results, suggests that the simple binary notion of high-spin and low-spin states carried by Ni^2+^ ions in these phases is likely a simplification. This shows that further investigations on single crystal samples are merited to enable the subtleties of these compounds to be fully accounted for.

## Supporting Information Available

Further diffractograms; refinement parameters; and computational results and physical property measurement data (PDF)
