# Supplementary material for: High- versus Low-Spin Ni2+ in Elongated Octahedral Environments: Sr2NiO2Cu2Se2, Sr2NiO2Cu2S2, and Sr2NiO2Cu2(Se1–xSx)2
Source: Chem Mater. 2022 Oct 18;34(21):9503–16. doi: 10.1021/acs.chemmater.2c02002 (PMC9648177; doi:10.1021/acs.chemmater.2c02002)
Supplement: Supplementary file 1 — cm2c02002_si_001.pdf [file cm2c02002_si_001.pdf]

High- vs. low-spin Ni^{2+} in elongated octahedral environments: $Sr_2NiO_2Cu_2Se_2$, $Sr_2NiO_2Cu_2S_2$ and $Sr_2NiO_2Cu_2(Se_{1-x}S_x)_2$

Robert D. Smyth,[a] Jack N. Blandy,[a,b] Ziyu Yu,[a] Shuai Liu,[a,c] Craig V. Topping,[d] Simon J. Cassidy,[a] Catherine F. Smura,[a] Daniel N. Woodruff,[a] Pascal Manuel,[e] Craig L. Bull,[e,f] Nicholas P. Funnell,[e] Christopher J. Ridley,[e] John E. McGrady,[a] and Simon J. Clarke[a,]*

[a]*Department of Chemistry, University of Oxford, Inorganic Chemistry Laboratory, South Parks Road, Oxford OX1 3QR, United Kingdom*

[b]*Diamond Light Source Ltd., Harwell Science and Innovation Campus, Didcot OX11 0DE, United Kingdom*

[c]*College of Chemistry and Chemical Engineering, Anhui University, Hefei 230601, Peoples Republic of China*

[d]*Department of Physics, University of Oxford, Clarendon Laboratory, Parks Road, Oxford OX1 3PU, United Kingdom*

[e]*ISIS Facility, Rutherford Appleton Laboratory Harwell Oxford, Didcot, OX1 10QX, United Kingdom*

[f]*School of Chemistry, The University of Edinburgh, King's Buildings, David Brewster Road, Edinburgh EH9 3FJ, U.K*

Supporting Information

* Corresponding author

email address: simon.clarke@chem.ox.ac.uk

Table of Contents

Figure S1. Rietveld refinement of $Sr_2NiO_2Cu_2(Se_{0.375}S_{0.625})_2$, showing the impurities from using a high temperature synthesis for the more S rich compounds in the $Sr_2NiO_2Cu_2(Se_{1-x}S_x)_2$ series of compounds. R_{wp} = 6.209 %; χ^2 = 1.422
Figure S2. Magnetisation *vs* field plot for $Sr_2NiO_2Cu_2Se_2$ after using a higher heating cycle.
Figure S3. Rietveld plot of a sample of "$Sr_2NiO_2Cu_2Se_2$" synthesised at 500 °C. The comparatively low synthesis temperature resulted in a highly impure sample. Data measured using the PANalytical X'Pert. R_{wp} = 11.082 %; χ^2 = 1.717
Figure S4. Rietveld plot of $Sr_2NiO_2Cu_2Se_2$ (JNB267B) measured at 300 K using the backscattering (168°) bank of HRPD. R_{wp} = 5.409 %; χ^2 = 1.051
Figure S5. DOS of Ni in $Sr_2NiO_2Cu_2S_2$ (Left) U = 0 (square planar) (Right) U = 4 (octahedral).
Figure S6. DOS of Ni in $Sr_2NiO_2Cu_2Se_2$ (Left) U = 0 (square planar) (Right) U = 4 (octahedral).
Figure S7. NPD patterns for $Sr_2NiO_2Cu_2Se_2$ measured on OSIRIS. Magnetic Bragg peaks (*) and peaks from the Al-tailed cryostat (#) are indicated.
Figure S8. Schematic of the individual effects of the two magnetic symmetry modes activated in the antiferromagnetic ordering model of $Sr_2NiO_2Cu_2Se_2$ (Table S1). For clarity only the Nickel atoms are shown.
Figure S9. Annotated powder neutron diffraction pattern of $Sr_2NiO_2Cu_2Se_2$, measured using OSIRIS at 5 K. The magnetic peaks are indexed using the expanded magnetic cell and the colour coding indicates which peaks are modelled by the $mX3^+$(a) mode (k-vector: ((½, ½, 0)) or the mP4(a) mode (k-vector: (½, ½, ½)).
Figure S10. Integrated intensity of the five largest magnetic peaks as a function of temperature, shown here both as all five peaks summed and separated into the peaks modelled by the $mX3^+$(a) mode (k-vector: ((½, ½, 0)) or the mP4(a) mode (k-vector: (½, ½, ½)). The intensity of the peaks was normalised with respect to the (008) nuclear peak (indexed using the expanded magnetic cell). The line is a guide to the eye.
Figure S11. First order derivative χ'_{mol} for $Sr_2NiO_2Cu_2Se_2$. Inset. χ'_{mol} for $Sr_2NiO_2Cu_2Se_2$ around the onset of magnetic ordering.
Figure S12. Magnetic susceptibility of $Sr_2NiO_2Cu_2Se_2$ (JNB231), measured using a 4 – 3 T subtraction compared with the magnetic susceptibility of $Sr_2NiO_2Cu_2S_2$ (obtained from ref. 45).
Figure S13. An observed kink in the *c* / *a* ratio of $Sr_2NiO_2Cu_2Se_2$ at 3.2 GPa during run A which lead to further investigation during run B. The error bars are within the points of the plot.
Figure S14. An observed kink in the Ni-Se bond length $Sr_2NiO_2Cu_2Se_2$ at 4GPa during run B.
Figure S15. The low pressure behaviour of the Ni-O bond lengths in $Sr_2NiO_2Cu_2Se_2$ during run B, this behaviour indicated there was no change in spin state and from that we were able to rule out that there was any anomaly and that Figures S11-S12 were artefacts of the experiment.
Figure S16. Changes in the *c* / *a* ratio as a function of pressure during run C, a change to the low-spin state would have led to an increased c / a ratio at the transition.
Figure S17. Changes in the Ni-Se bond lengths as a function of pressure using sintered diamond anvils during run C.
Figure S18. Variation with pressure of the unit cell volume for $Sr_2NiO_2Cu_2Se_2$ for the high pressure region. The solid line is the fit to the data using a third-order Birch-Murnaghan EoS.
Figure S19. Variation with pressure of the *a* lattice parameter cubed for $Sr_2NiO_2Cu_2Se_2$ for the low pressure region. The solid line is the fit to the data using a third-order Birch-Murnaghan EoS.
Figure S20. Variation with pressure of the *c* lattice parameter cubed for $Sr_2NiO_2Cu_2Se_2$ for the low pressure region. The solid line is the fit to the data using a third-order Birch-Murnaghan EoS.
Figure S21. Changes in the Ni-O bond length across the series.
Figure S22. Changes in the Ni-Ch bond length across the series.
Figure S23. Changes in the *c* / *a* ratio as a function of temperature for $Sr_2NiO_2Cu_2(Se_{1-x}S_x)_2$ $0 \le x \le 0.5$.

Figure S24. Changes in the Ni-O bond length as a function of temperature for $Sr_2NiO_2Cu_2(Se_{1-x}S_x)_2$ $0 \le x \le 0.5$.
Figure S25. SQUID data for the $Sr_2NiO_2Cu_2(Se_{1-x}S_x)_2$ series of compounds for $0 \le x \le 0.5$
Figure S26. Rietveld refinement at 1.5 K of $Sr_2NiO_2Cu_2(Se_{0.75}S_{0.25})_2$ against bank 2/9 of the WISH diffractometer at ISIS. R_{wp} = 5.494 %; χ^2 = 0.027
Figure S27. Rietveld refinement at 1.5 K of $Sr_2NiO_2Cu_2(Se_{0.625}S_{0.375})_2$ against bank 2/9 of the WISH diffractometer at ISIS. R_{wp} = 5.077 %; χ^2 = 0.028
Figure S28. Rietveld refinement at 1.5 K of $Sr_2NiO_2Cu_2(Se_{0.5}S_{0.5})_2$ against bank 2/9 of the WISH diffractometer at ISIS. R_{wp} = 5.051 %; χ^2 = 0.013
Figure S29. The appearance of a highly asymmetric peak on cooling in $Sr_2NiO_2Cu_2(Se_{0.65625}S_{0.34375})_2$ which can be modelled using a Warren function. The peak above 6 Å is rather an experimental artefact or a miniscule amount of an unidentified impurity.
Figure S30. The function used and resulting parameters to model the Warren peak.[1–3]
Table S1. Comparison of structural parameters for $Sr_2MO_2Cu_2S_2$ (*M* = Co, Ni, Cu, Zn).
Table S2. Structure data by experiment and calculation for $Sr_2MO_2Cu_2Ch_2$.
Table S3. Computational data for $Sr_2NiO_2Cu_2S_2$ using different U values for Ni.
Table S4. Calculation data of $Sr_2NiO_2Cu_2Se_2$ using different U values for Ni.
Table S5. Refined magnetic moments of Ni atoms in $Sr_2NiO_2Cu_2Se_2$; data obtained by combined refinement against PND data of $Sr_2NiO_2Cu_2Se_2$ (JNB267B) measured at 5 K using OSIRIS and HRPD. $P_C4_12_12$ (92.117 in the Belov-Neronova-Smirnova (BNS) scheme).
Table S6. Refined parameters for $Sr_2NiO_2Cu_2Se_2$ from PEARL.
Table S7. Structural Model for $Sr_2NiO_2Cu_2(Se_{1-x}S_x)_2$ ($0.125 \le x \le 0.5$).
Table S8. Refined atomic coordinates for $Sr_2NiO_2Cu_2(Se_{1-x}S_x)_2$ ($0.125 \le x \le 0.5$) from PND.
Table S9. Refined U_{iso} parameters for $Sr_2NiO_2Cu_2(Se_{1-x}S_x)_2$ ($0.125 \le x \le 0.5$) from PXRD at RT.
Table S10. Refined U_{iso} parameters for $Sr_2NiO_2Cu_2(Se_{1-x}S_x)_2$ ($0.125 \le x \le 0.5$) from PND at RT.
Table S11. Comparison of structural parameters for $Sr_2NiO_2Cu_2(Se_{1-x}S_x)_2$ ($0.125 \le x \le 0.5$) at RT.
Appendix i. Alternative model for computation of crystal and electronic structures.

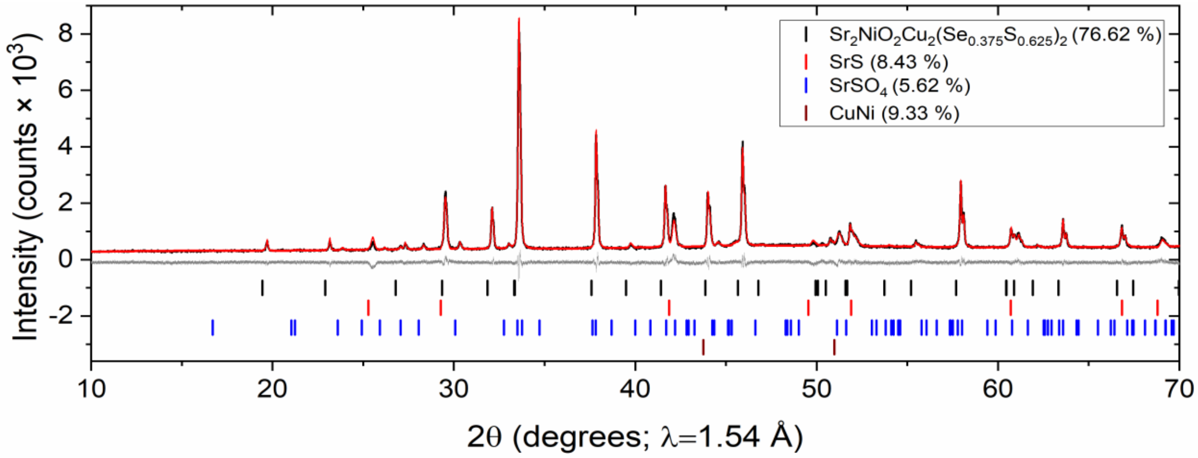

Figure S1. Rietveld refinement of $Sr_2NiO_2Cu_2(Se_{0.375}S_{0.625})_2$, showing the impurities from using a high temperature synthesis for the more S rich compounds in the $Sr_2NiO_2Cu_2(Se_{1-x}S_x)_2$ series of compounds. R_{wp} = 6.209 %; χ^2 = 1.422

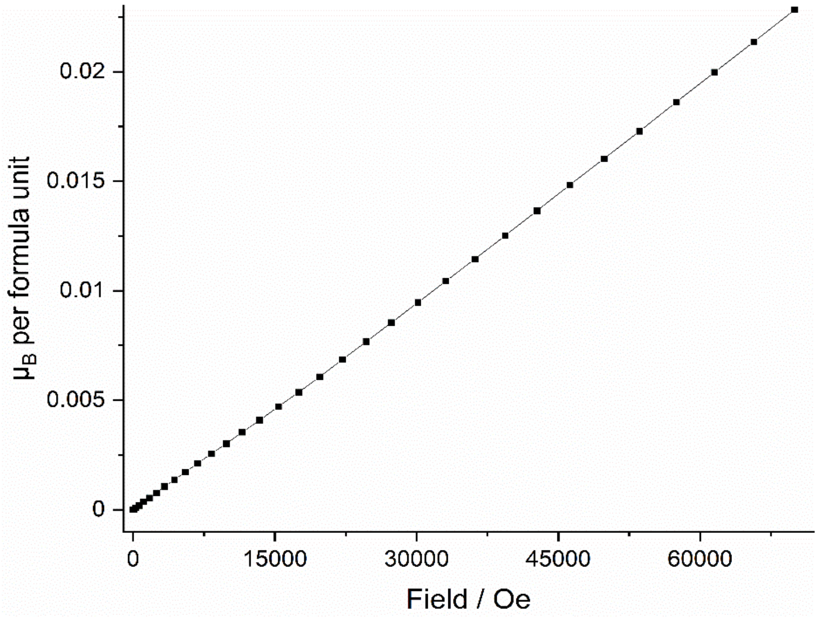

Figure S2. Magnetisation *vs* field plot for $Sr_2NiO_2Cu_2Se_2$ after using a higher temperature heating cycle during synthesis.

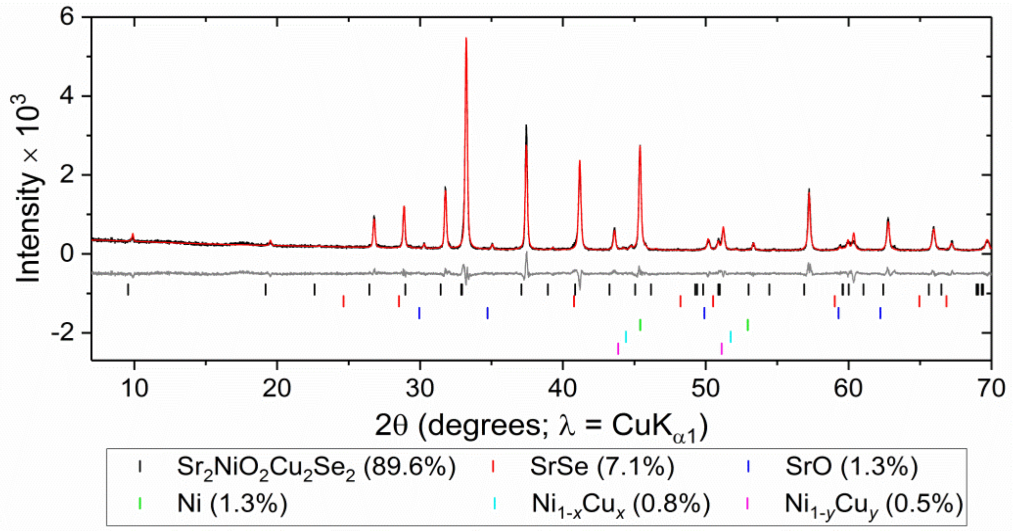

Figure S3. Rietveld plot of a sample of "$Sr_2NiO_2Cu_2Se_2$" synthesised at 500 °C. The comparatively low synthesis temperature resulted in a highly impure sample. Data measured using the PANalytical X'Pert. R_{wp} = 11.082 %; χ^2 = 1.717

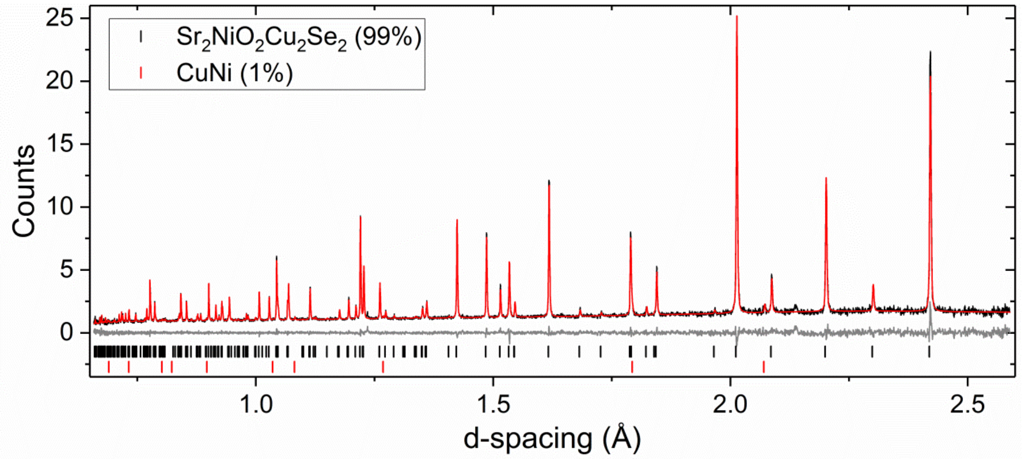

Figure S4. Rietveld plot of $Sr_2NiO_2Cu_2Se_2$ (JNB267B) measured at 300 K using the backscattering (168°) bank of HRPD. R_{wp} = 5.409 %; χ^2 = 1.051

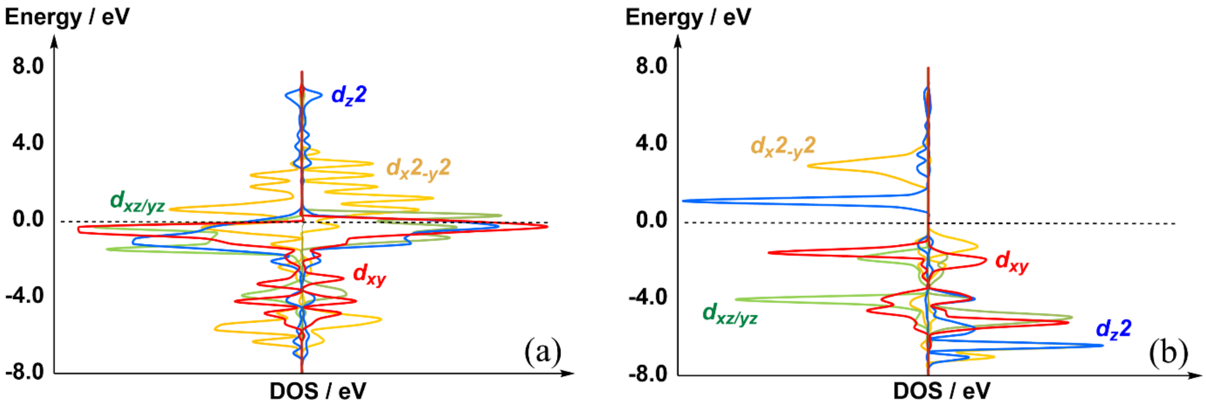

Figure S5. DOS of Ni in $Sr_2NiO_2Cu_2S_2$ (Left) U = 0 (square planar) (Right) U = 4 (octahedral).

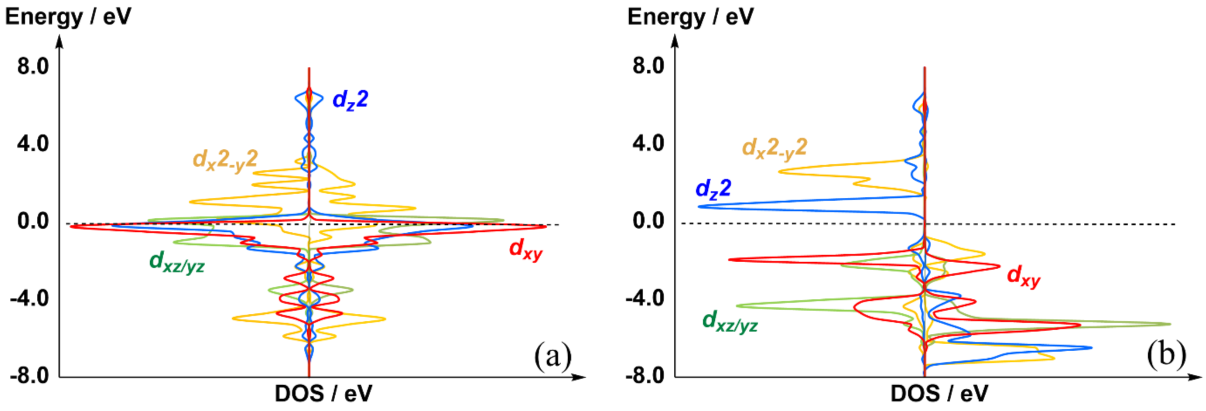

Figure S6. DOS of Ni in $Sr_2NiO_2Cu_2Se_2$ (Left) U = 0 (square planar) (Right) U = 4 (octahedral).

Magnetic Bragg peaks as a function of temperature from OSIRIS data. Only a small segment of the diffraction pattern was collected at 120 K, 140 K and 160 K due to the limited availability of instrument time. This small segment (4.1 ≤ *d* (Å) ≤ 6.2) contains five magnetic peaks, but only one structural peak. This meant that when attempts were made to refine the magnetic structure in order to obtain the ordered moment as a function of temperature, the calculated errors were unacceptably large. Therefore, the peak intensity of the five magnetic peaks has been integrated as a proxy for the magnetic moment, as shown in Figure S10. The integrated intensity of the peaks that can be indexed using the k-vectors (½, ½, 0) (modelled using the $mX3^+$(a) mode) and (½, ½, ½) (modelled using the mP4(a) mode) have also been integrated separately so changes in the relationship of the magnetic moment in the *ab*-plane ($mX3^+$(a) mode) and the *c*-axis (mP4(a) mode) can be observed. For clarity, Figure S9. shows which peaks are being referred to.

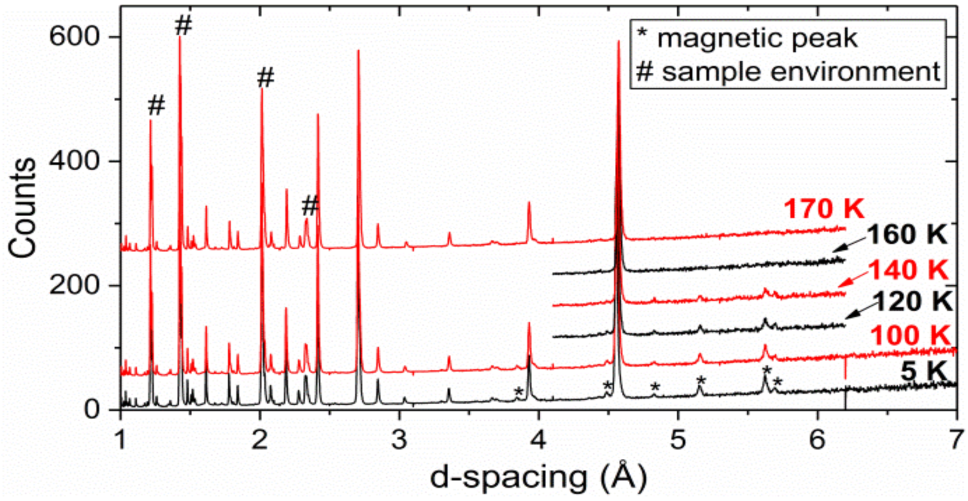

Figure S7. NPD patterns for $Sr_2NiO_2Cu_2Se_2$ measured on OSIRIS. Magnetic Bragg peaks (*) and peaks from the Al-tailed cryostat (#) are indicated.

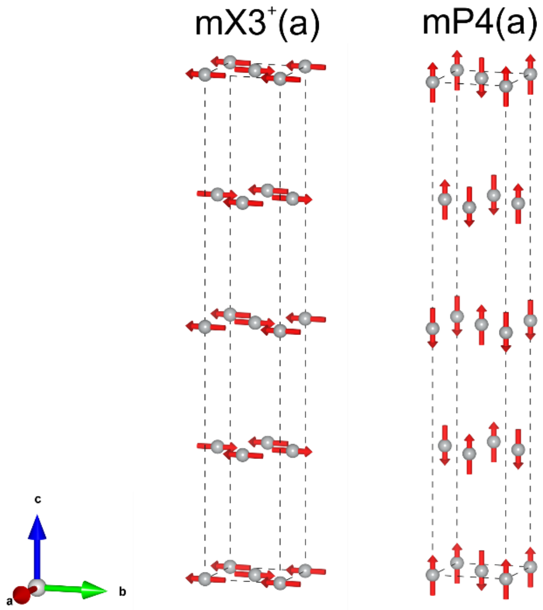

Figure S8. Schematic of the individual effects of the two magnetic symmetry modes activated in the antiferromagnetic ordering model of $Sr_2NiO_2Cu_2Se_2$ (Table S1). For clarity only the Nickel atoms are shown.

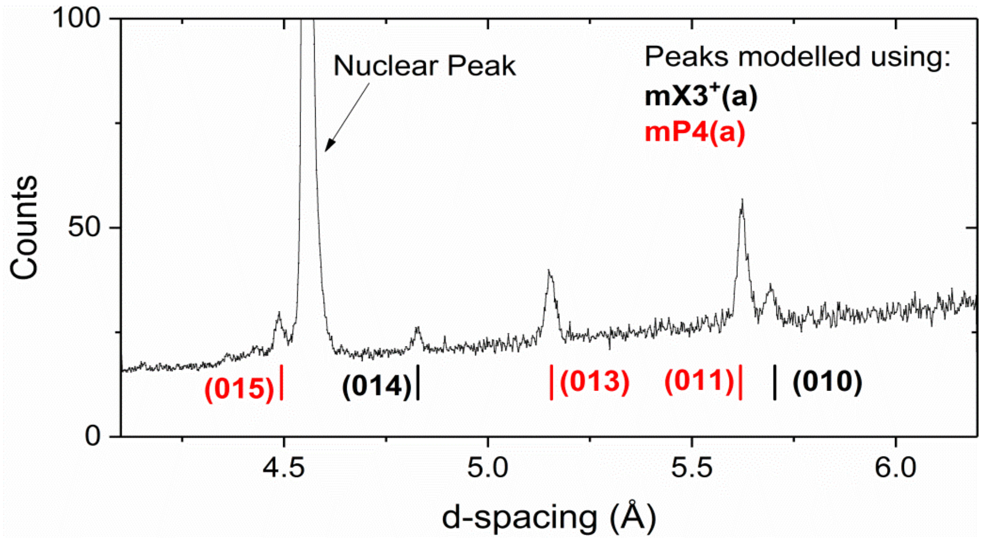

Figure S9. Annotated powder neutron diffraction pattern of $Sr_2NiO_2Cu_2Se_2$, measured using OSIRIS at 5 K. The magnetic peaks are indexed using the expanded magnetic cell and the colour coding indicates which peaks are modelled by the $mX3^+$(a) mode (k-vector: ((½, ½, 0)) or the mP4(a) mode (k-vector: (½, ½, ½)).

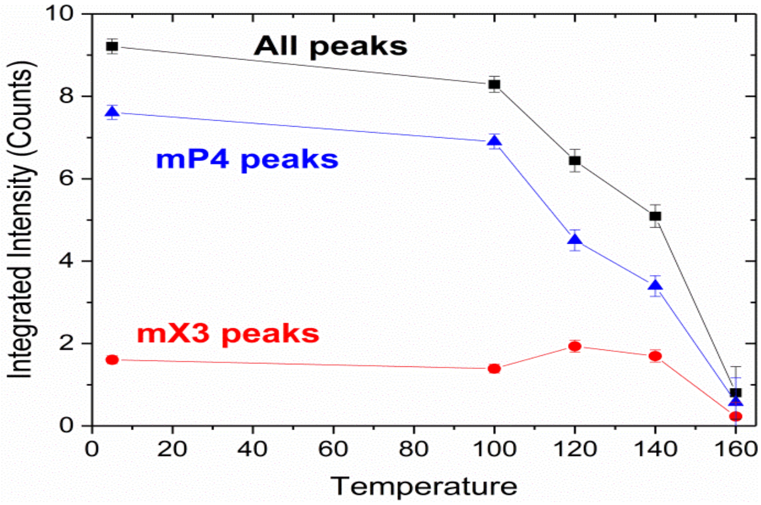

Figure S10. Integrated intensity of the five largest magnetic peaks as a function of temperature, shown here both as all five peaks summed and separated into the peaks modelled by the mX3$^+$(a) mode (k-vector: ((½, ½, 0)) or the mP4(a) mode (k-vector: (½, ½, ½)). The intensity of the peaks was normalised with respect to the (008) nuclear peak (indexed using the expanded magnetic cell). The line is a guide to the eye.

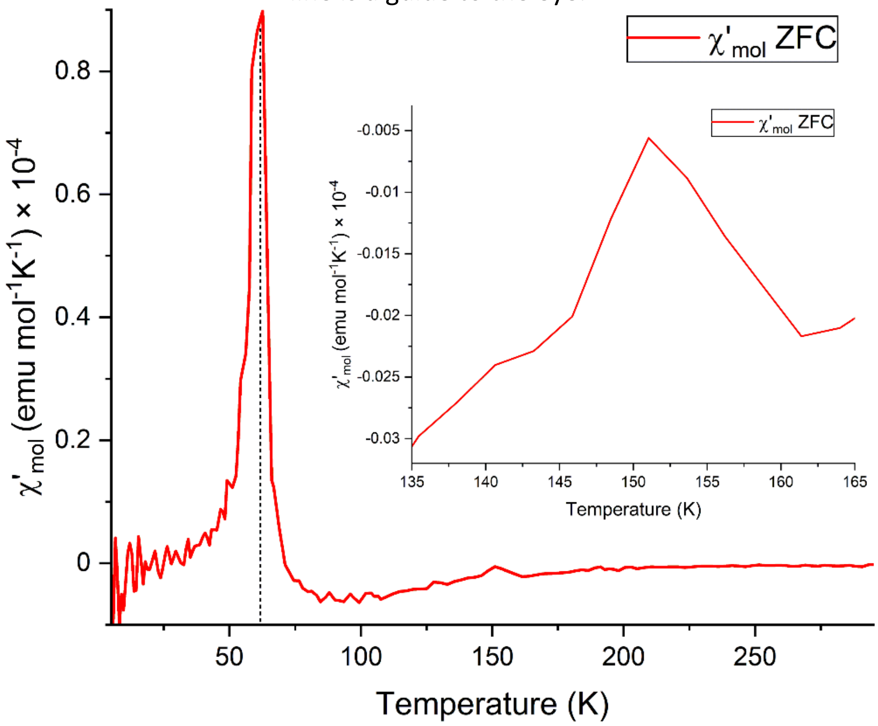

Figure S11. First order derivative χ'_{mol} for $Sr_2NiO_2Cu_2Se_2$. Inset. χ'_{mol} for $Sr_2NiO_2Cu_2Se_2$ around the onset of magnetic ordering.

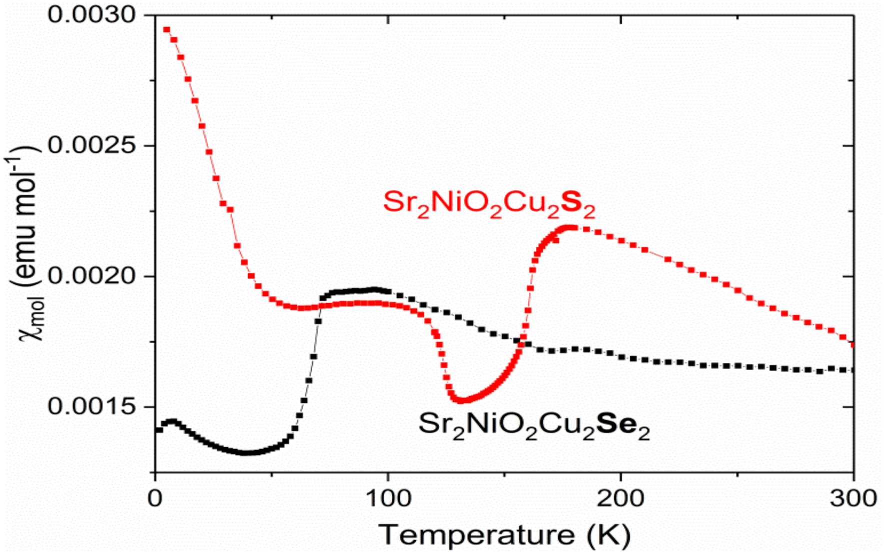

Figure S12. Magnetic susceptibility of $Sr_2NiO_2Cu_2Se_2$ (JNB231), measured using a 4 – 3 T subtraction compared with the magnetic susceptibility of $Sr_2NiO_2Cu_2S_2$ (obtained from ref. 45).

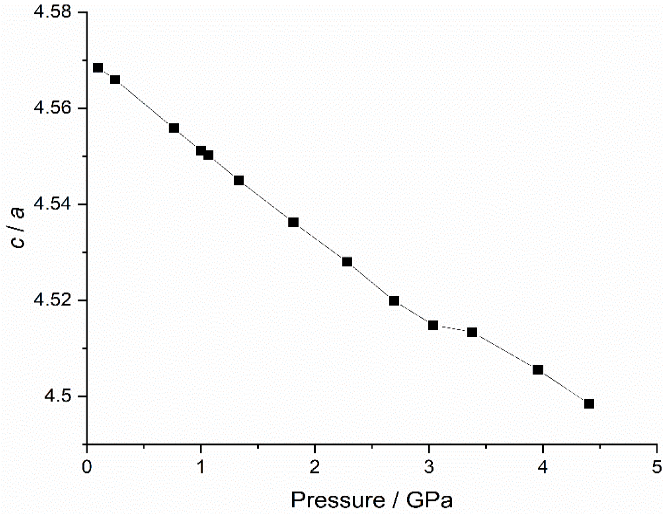

Figure S13. An observed kink in the *c* / *a* ratio of $Sr_2NiO_2Cu_2Se_2$ at 3.2 GPa during run A which lead to further investigation during run B. The error bars are within the points of the plot.

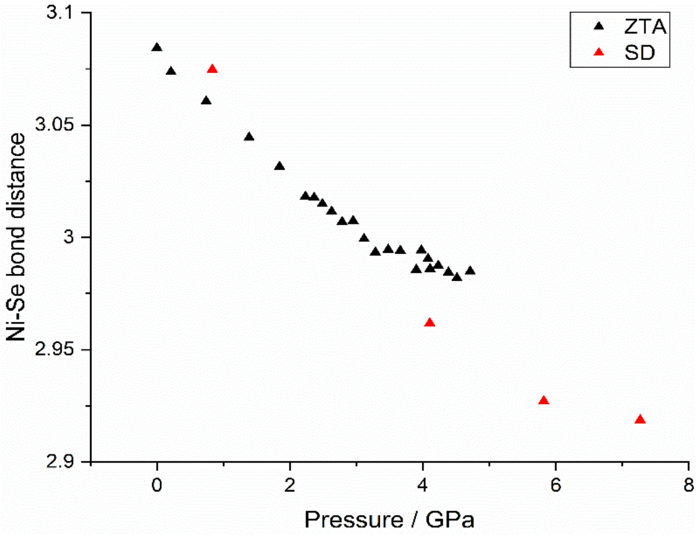

Figure S14. An observed kink in the Ni-Se bond length $Sr_2NiO_2Cu_2Se_2$ at 4GPa during run B.

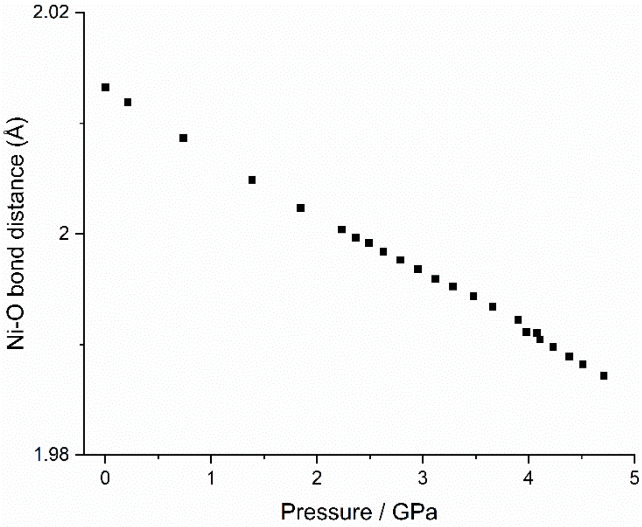

Figure S15. The low pressure behaviour of the Ni-O bond lengths in $Sr_2NiO_2Cu_2Se_2$ during run B, this behaviour indicated there was no change in spin state and from that we were able to rule out that there was any anomaly and that Figures S11-S12 were artefacts of the experiment.

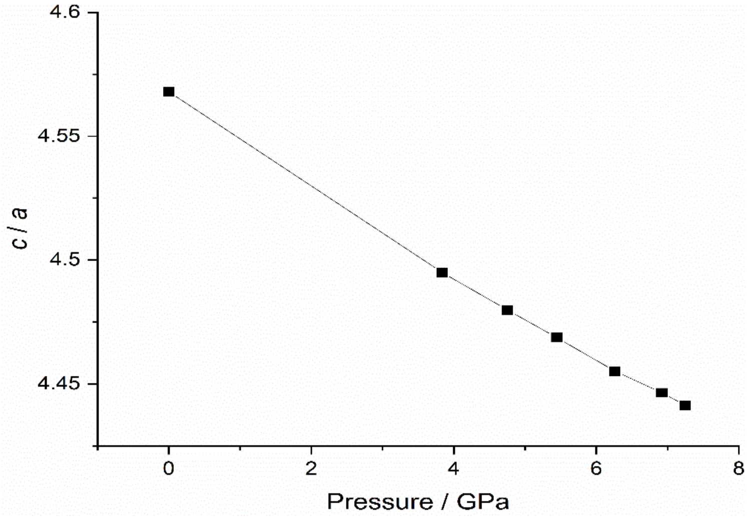

Figure S16. Changes in the *c* / *a* ratio as a function of pressure during run C, a change to the low-spin state would have led to an increased c / a ratio at the transition.

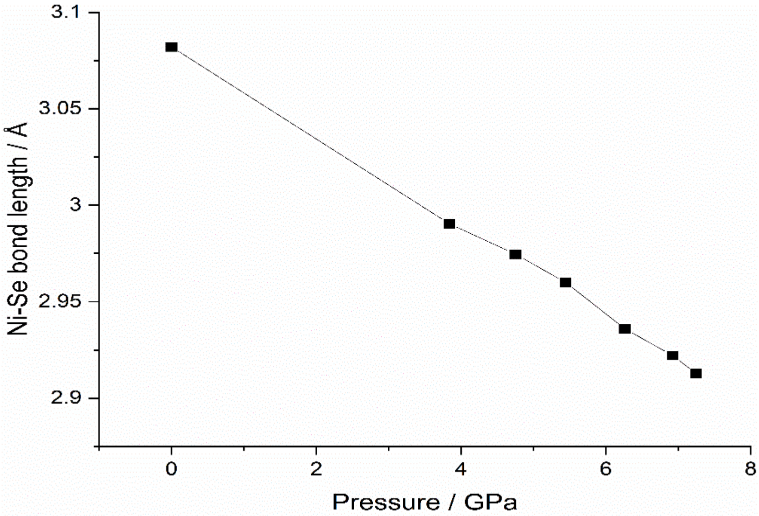

Figure S17. Changes in the Ni-Se bond lengths as a function of pressure using sintered diamond anvils during run C.

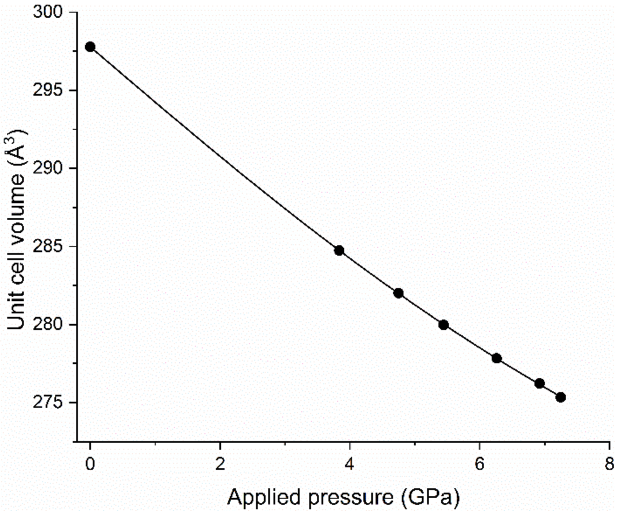

Figure S18. Variation with pressure of the unit cell volume for $Sr_2NiO_2Cu_2Se_2$ for the high pressure region. The solid line is the fit to the data using a third-order Birch-Murnaghan EoS.

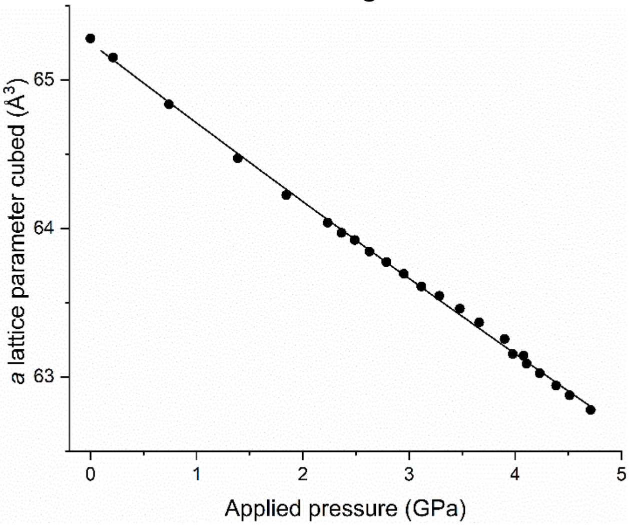

Figure S19. Variation with pressure of the *a* lattice parameter cubed for $Sr_2NiO_2Cu_2Se_2$ for the low pressure region. The solid line is the fit to the data using a third-order Birch-Murnaghan EoS.

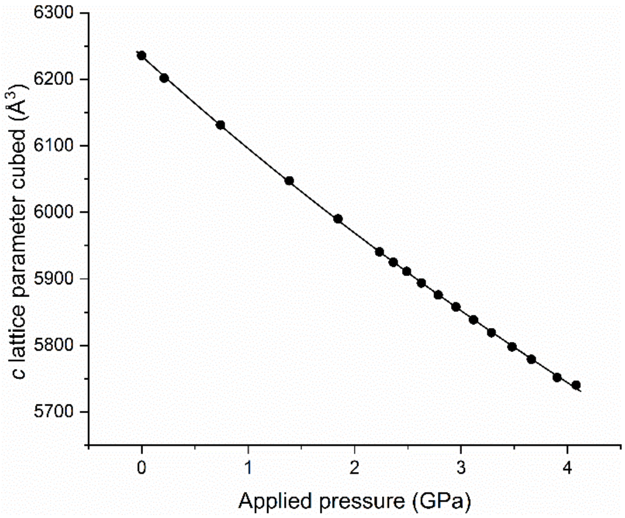

Figure S20. Variation with pressure of the *c* lattice parameter cubed for $Sr_2NiO_2Cu_2Se_2$ for the low pressure region. The solid line is the fit to the data using a third-order Birch-Murnaghan EoS.

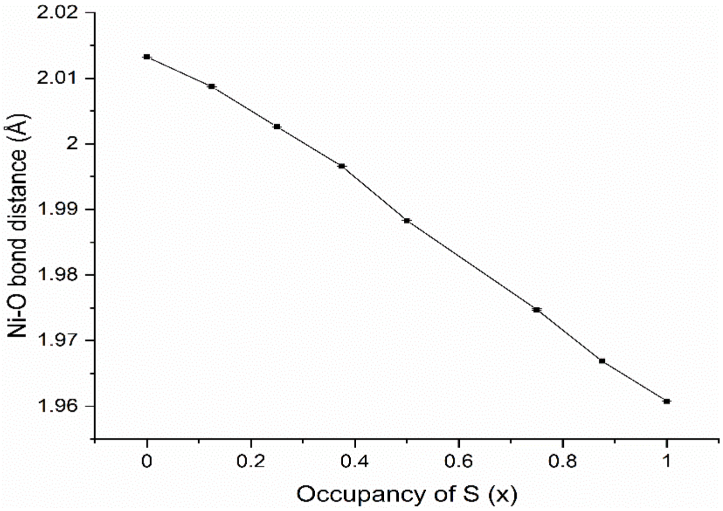

Figure S21. Changes in the Ni-O bond length across the series.

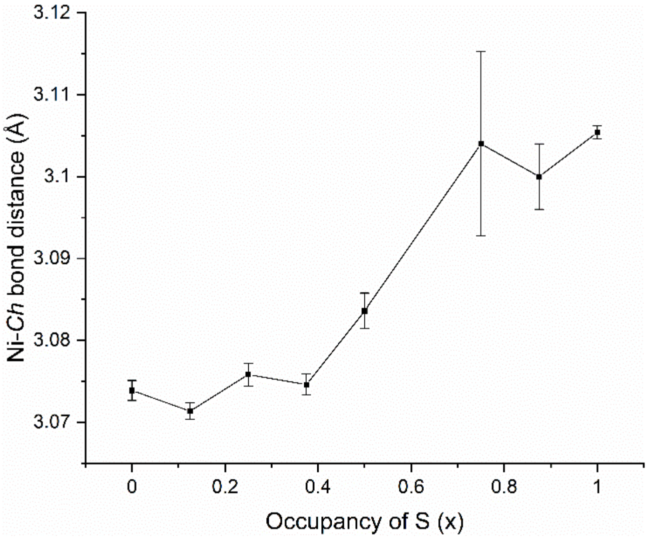

Figure S22. Changes in the Ni-Ch bond length across the series.

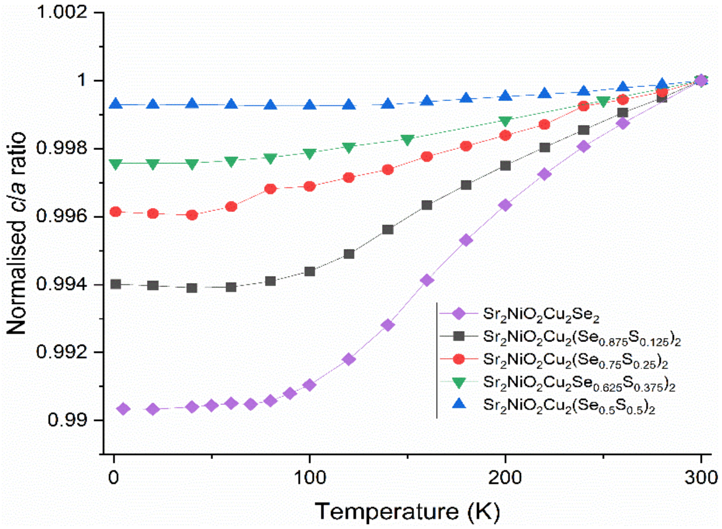

Figure S23. Changes in the *c* / *a* ratio as a function of temperature for $Sr_2NiO_2Cu_2(Se_{1-x}S_x)_2$ $0 \leq x \leq 0.5$.

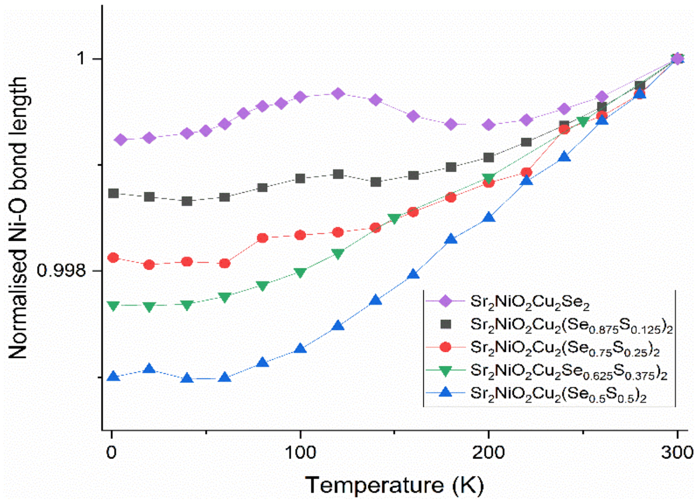

Figure S24. Changes in the Ni-O bond length as a function of temperature for $Sr_2NiO_2Cu_2(Se_{1-x}S_x)_2$ $0 \leq x \leq 0.5$.

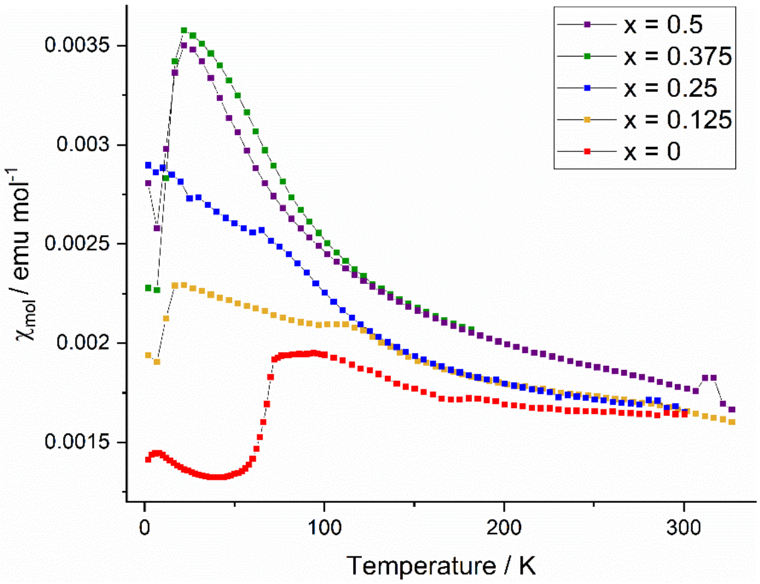

Figure S25. SQUID data for the $Sr_2NiO_2Cu_2(Se_{1-x}S_x)_2$ series of compounds for $0 \leq x \leq 0.5$

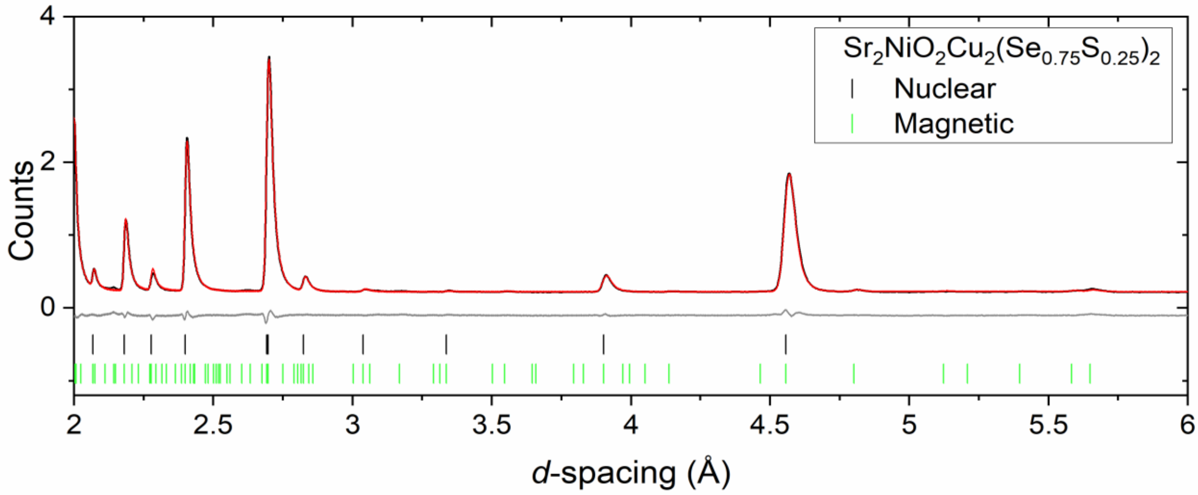

Figure S26. Rietveld refinement at 1.5 K of $Sr_2NiO_2Cu_2(Se_{0.75}S_{0.25})_2$ against bank 2/9 of the WISH diffractometer at ISIS. R_{wp} = 5.494 %; χ^2 = 0.027

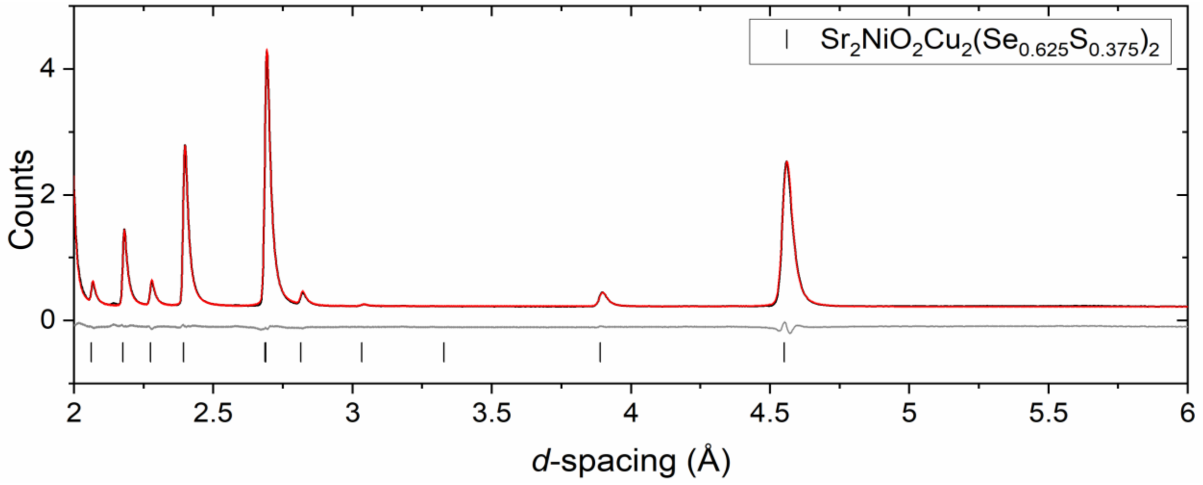

Figure S27. Rietveld refinement at 1.5 K of $Sr_2NiO_2Cu_2(Se_{0.625}S_{0.375})_2$ against bank 2/9 of the WISH diffractometer at ISIS. R_{wp} = 5.077 %; χ^2 = 0.028

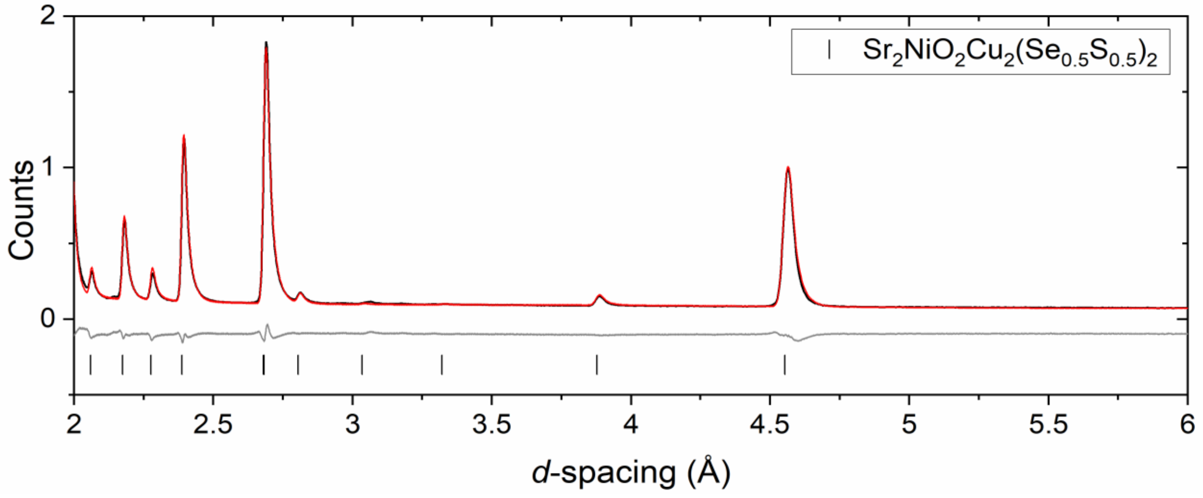

Figure S28. Rietveld refinement at 1.5 K of $Sr_2NiO_2Cu_2(Se_{0.5}S_{0.5})_2$ against bank 2/9 of the WISH diffractometer at ISIS. R_{wp} = 5.051 %; χ^2 = 0.013

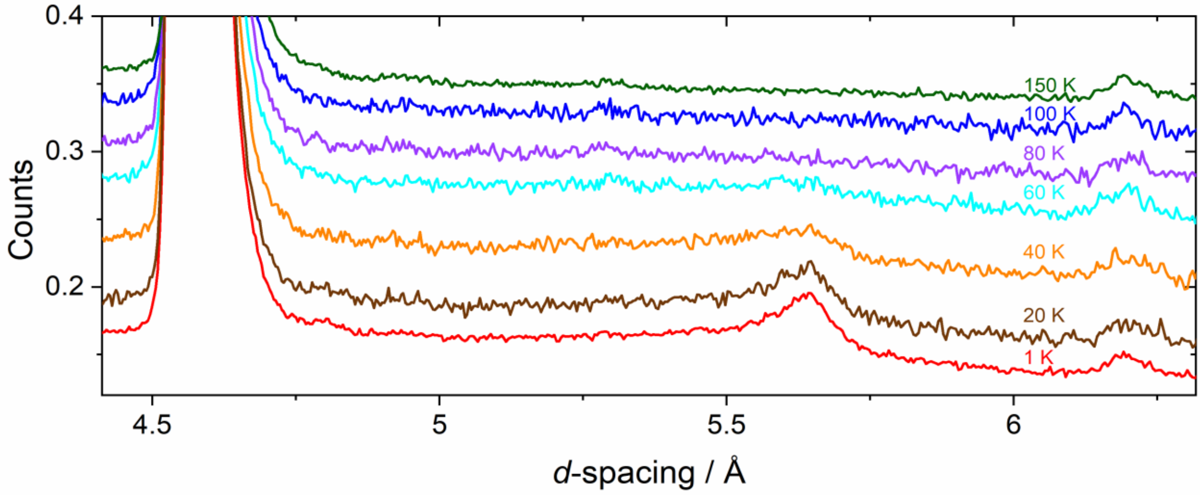

Figure S29. The appearance of a highly asymmetric peak on cooling in $Sr_2NiO_2Cu_2(Se_{0.65625}S_{0.34375})_2$ which can be modelled using a Warren function. The peak above 6 Å is rather an experimental artefact or a miniscule amount of an unidentified impurity.

$$S(\boldsymbol{Q}) = \frac{1}{2\pi\sigma_{\perp}^{2}} \exp[-\frac{1}{2}\left(\frac{|\boldsymbol{Q}-\tau|_{\perp}}{\sigma_{\perp}}\right)^{2}] \frac{X_i}{\pi} \frac{1}{1+(X_i|\boldsymbol{Q}-\tau|_{\parallel})^{2}}$$

Model	Warren-like
σ	0.0658 ± 0.0031
τ_1	1.21417 ± 0.00769
τ_2	1.1136 ± 2.45567E-4
X_i	97.81477 ± 5.07122
Reduced χ^2	7.5416E-6
R-Square (COD)	0.96841
Adj. R-Square	0.96774

Figure S30. The function used and resulting parameters to model the Warren peak.[1–3]

The above function was used to model the additional peak in Figure 14 from the main text that was identified as being Warren-like. The Gaussian and Lorentzian parts describe the long-range in-plane correlation and short-range correlation along lattice parameter *c* respectively. $\sigma_{\perp}$ represents the in-plane peak width, X_i is the correlation-length and τ is the position of the centre of the peak.

Table S1. Comparison of structural parameters for $Sr_2MO_2Cu_2S_2$ (*M* = Co, Ni, Cu, Zn).

Compound	$Sr_2CoO_2Cu_2S_2$	$Sr_2NiO_2Cu_2S_2$	$Sr_2CuO_2Cu_2S_2$	$Sr_2ZnO_2Cu_2S_2$
Reference	ref.[4]	ref.[5]	ref.[6]	ref.[7,8]
Radiation	PND	PND	PND	X-Ray
a (Å)	3.99129(2)	3.92159(2)	3.92016(6)	4.01084(3)
c (Å)	17.71555(9)	18.11558(15)	18.2012(4)	17.7360(2)
c/a	4.43855(3)	4.61945(4)	4.64297(12)	4.4222(4)
Volume (Å^3)	282.216(3)	278.597(5)	279.71(1)	284.63(2)
M–O (Å)	1.99565(1)	1.96080(1)	1.96008(3)	2.0059(1)
M–S (Å)	3.0327(5)	3.1054(8)	3.1085(15)	3.051(1)
M–S/*M*–O	1.5197(3)	1.5837(4)	1.5859(8)	1.5210(6)
S–Cu–S (Å)	2.4379(5)	2.4230(5)	2.43094(4)	2.450(3)
S–Cu–S, α (°) [2]	109.36(2)	108.04(3)	107.4732(15)	109.31(7)
Se–Cu–S, β (°) [4]	109.68(3)	110.191(15)	110.4794(8)	109.79(14)

Table S2. Structure data by experiment and calculation for $Sr_2MO_2Cu_2Ch_2$.

	$Sr_2CoO_2Cu_2S_2$		$Sr_2CoO_2Cu_2Se_2$	
	exp	DFT	exp	DFT
a / Å	5.643	5.617	5.720	5.705
c / Å	17.720	17.460	18.357	18.102
Co-O / Å	1.986	1.986	2.024	2.017
Co-E / Å	3.008	3.007	3.0827	3.041
Co-E/Co-O	1.515	1.514	1.523	1.507
μ(Co)		2.60		2.60

	$Sr_2CuO_2Cu_2S_2$		$Sr_2CuO_2Cu_2Se_2$	
	exp	DFT	exp	DFT
a / Å	5.544	5.509	5.616	5.610
c / Å	18.20	18.034	18.820	18.604
Cu-O / Å	1.96	1.948	1.986	1.984
Cu-E / Å	3.11	3.115	3.169	3.143
Cu-E/Cu-O	1.587	1.599	1.596	1.584
μ(Cu)		0.90		0.90

Table S3. Computational data for $Sr_2NiO_2Cu_2S_2$ using different U values for Ni.

U = Ni^{2+}		Energy / eV	ΔE / eV	Spin density	d-orbital configuration	M-O / Å	M-S / Å	*a* / Å	*c* / Å
	Exp					1.96	3.11	3.92	18.12
4	Cal	-177.67	-0.97	1.61,-1.61,1.61,-1.61	Octahedral	1.99	3.03	3.98	17.60
4	Cal	-176.69		0,0,0,0	Square-planar	1.93	3.21	3.86	18.52
3	Cal	-179.07	-0.36	1.55,-1.55,1.55,-1.55	Octahedral	1.99	3.03	3.98	17.61
3	Cal	-178.71		0,0,0,0	Square-planar	1.93	3.22	3.85	18.58
2	Cal	-180.69	0.14	1.47,-1.47,1.47,-1.47	Octahedral	1.99	3.01	3.99	17.49
2	Cal	-180.86		0.37,0.37,0.37,0.37	Square-planar	1.94	3.21	3.87	18.44
1	Cal	-183.09		0,0,0,0	Square-planar	1.93	3.21	3.86	18.47
0	Cal	-185.71		-0.51,-0.51,-0.51,-0.51	Square-planar	1.93	3.20	3.87	18.44

$\Delta E = E_{oct} - E_{squ}$

Table S4. Calculation data of $Sr_2NiO_2Cu_2Se_2$ using different U values for Ni.

U = Ni^{2+}		Energy / eV	ΔE / eV	Spin density	d-orbital configuration	M-O / Å	M-S / Å	*a* / Å	*c* / Å
	Exp					2.01	3.08	4.02	18.39
4	Cal	-171.60	-1.70	1.608,-1.608,1.608, -1.608	Octahedral	2.03	3.05	4.05	18.17
4	Cal	-169.90		-0.005,-0.005,0.005, 0.005	Square-planar	1.97	3.22	3.94	18.98
3	Cal	-173.02	-0.90	1.548,-1.548,1.548, -1.548	Octahedral	2.03	3.05	4.05	18.18
3	Cal	-172.12		0.363,0.363,-0.363, -0.363	Square-planar	1.97	3.24	3.95	19.03
2	Cal	-174.58	-0.32	1.471,-1.471,1.471, -1.471	Octahedral	2.02	3.05	4.05	18.17
2	Cal	-174.26		-0.352,-0.352,0.352, 0.352	Square-planar	1.97	3.24	3.95	19.03
1	Cal	-176.53		0.534,0.536,-0.534, -0.536	Square-planar	1.97	3.23	3.95	19.03
1	Cal	-176.54		0.546, 0.546, 0.546, 0.546	Square-planar	1.98	3.21	3.96	18.89
0	Cal	-179.03		0.508,0.508,0.508, 0.508	Square-planar	1.97	3.22	3.95	18.98
0	Cal	-178.98		-0.504,0.504,0.504, 0.504	Square-planar	1.98	3.21	3.96	18.91

$\Delta E = E_{oct} - E_{squ}$

Table S5. Refined magnetic moments of Ni atoms in $Sr_2NiO_2Cu_2Se_2$; data obtained by combined refinement against PND data of $Sr_2NiO_2Cu_2Se_2$ (JNB267B) measured at 5 K using OSIRIS and HRPD. $P_C4_12_12$ (92.117 in the Belov-Neronova-Smirnova (BNS) scheme).

	μ_a (μ_B)	μ_b (μ_B)	μ_c (μ_B)	$\lvert\mu\rvert$ (μ_B)
Ni (0.75, 0.75, 0.75)	0.46(2)	0.46(2)	-1.14(2)	1.31(2)
R_{wp} (%)	4.461			
χ^2	1.322			

Table S6. Refined parameters for $Sr_2NiO_2Cu_2Se_2$ from PEARL.

	40 tonnes	50 tonnes	60 tonnes	70 tonnes	80 tonnes
p / GPa	3.84(5)	4.75(6)	5.39(8)	6.3(1)	6.9(1)
a / Å	3.98632(4)	3.97798(5)	3.97191(5)	3.96578(5)	3.96051(7)
c / Å	17.9193(4)	17.8217(4)	17.7511(4)	17.6731(4)	17.6104(6)
Ni-O / Å	1.99316(2)	1.98899(2)	1.98595(3)	1.98289(3)	1.98025(4)
c / *a*	4.4952(1)	4.4801(1)	4.4692(1)	4.4564(1)	4.4465(2)
Pb *v* / Å^3	112.67(4)	111.07(4)	110.03(4)	108.71(5)	107.76(6)

Table S7. Structural Model for $Sr_2NiO_2Cu_2(Se_{1-x}S_x)_2$ (0.125 ≤ *x* ≤ 0.5).

Atom	Position	*x*	*y*	*z*
Sr	4*e*	0	0	zSr
Ni	2*a*	0	0	0
Cu	4*c*	0	0.5	0.25
O	4*d*	0	0.5	0
Se	4*e*	0	0	*zCh*
S	4*e*	0	0	*zCh*
Space group = *I*4/*mmm*				

Table S8. Refined atomic coordinates for $Sr_2NiO_2Cu_2(Se_{1-x}S_x)_2$ (0.125 ≤ *x* ≤ 0.5) from PND.

	*z*Sr / RT	*zSr* / 1.5 K	*zCh* / RT	*zCh* / 1.5 K
$Sr_2NiO_2Cu_2(Se_{0.875}S_{0.125})_2$	0.41182(8)	0.41084(5)	0.16808(5)	0.16772(3)
$Sr_2NiO_2Cu_2(Se_{0.75}S_{0.25})_2$	0.4116(1)	0.41004(6)	0.16794(6)	0.16791(4)
$Sr_2NiO_2Cu_2(Se_{0.71875}S_{0.28125})_2$	-	0.40977(5)	-	0.16791(4)
$Sr_2NiO_2Cu_2(Se_{0.6875}S_{0.3125})_2$	-	0.40947(5)	-	0.16766(4)
$Sr_2NiO_2Cu_2(Se_{0.65625}S_{0.34375})_2$	-	0.41051(6)	-	0.16806(4)
$Sr_2NiO_2Cu_2(Se_{0.625}S_{0.375})_2$	0.41109(9)	0.41060(7)	0.16827(6)	0.16804(5)
$Sr_2NiO_2Cu_2(Se_{0.5}S_{0.5})_2$	0.4091(2)	0.4089(2)	0.16810(8)	0.16774(6)

Table S9. Refined U_{iso} parameters for $Sr_2NiO_2Cu_2(Se_{1-x}S_x)_2$ (0.125 ≤ *x* ≤ 0.5) from PXRD at RT.

	$Sr_2NiO_2Cu_2(Se_{0.875}S_{0.125})_2$	$Sr_2NiO_2Cu_2(Se_{0.75}S_{0.25})_2$	$Sr_2NiO_2Cu_2(Se_{0.625}S_{0.375})_2$	$Sr_2NiO_2Cu_2(Se_{0.5}S_{0.5})_2$
U_{iso}(Sr1) / Å^2	0.01008	0.0050(1)	0.00451(9)	0.0043(1)
U_{iso}(Ni1) / Å^2	0.01676	0.0059(2)	0.0057(2)	0.0062(2)
U_{iso}(Cu1) / Å^2	0.0123(2)	0.0180(2)	0.0163(2)	0.0174(2)
U_{iso}(O1) / Å^2	0.0017(7)	0.0039(7)	0.0064(6)	0.0037(6)
U_{iso}(*Ch*1) / Å^2	0.0041(1)	0.0054(2)	0.0063(1)	0.0071(2)

Table S10. Refined U_{iso} parameters for $Sr_2NiO_2Cu_2(Se_{1-x}S_x)_2$ (0.125 ≤ *x* ≤ 0.5) from PND at RT.

	$Sr_2NiO_2Cu_2(Se_{0.875}S_{0.125})_2$	$Sr_2NiO_2Cu_2(Se_{0.75}S_{0.25})_2$	$Sr_2NiO_2Cu_2(Se_{0.625}S_{0.375})_2$	$Sr_2NiO_2Cu_2(Se_{0.5}S_{0.5})_2$
U_{iso}(Sr1) / Å^2	0.0129(4)	0.0093(4)	0.0101(4)	0.0084(3)
U_{iso}(Ni1) / Å^2	0.0172(5)	0.0129(4)	0.0126(5)	0.0233(5)
U_{iso}(Cu1) / Å^2	0.0234(5)	0.0193(5)	0.0182(6)	0.0202(4)
U_{iso}(O1) / Å^2	0.0134(5)	0.0115(5)	0.0107(5)	0.0192(6)
U_{iso}(*Ch*1) / Å^2	0.0167(5)	0.0139(6)	0.0128(6)	0.0206(8)

Table S11. Comparison of structural parameters for $Sr_2NiO_2Cu_2(Se_{1-x}S_x)_2$ (0.125 ≤ *x* ≤ 0.5) at RT.

	$Sr_2NiO_2Cu_2(Se_{0.875}S_{0.125})_2$	$Sr_2NiO_2Cu_2(Se_{0.75}S_{0.25})_2$	$Sr_2NiO_2Cu_2(Se_{0.625}S_{0.375})_2$	$Sr_2NiO_2Cu_2(Se_{0.5}S_{0.5})_2$
Reference	This work	This work	This work	This work
Radiation	PND	PND	PND	PND
a (Å)	4.01790(4)	4.00594(4)	3.99345(3)	3.982128
c (Å)	18.3754(3)	18.3516(3)	18.3021(2)	18.290647
c/a	4.57338(8)	4.58111(9)	4.58304(7)	4.59318
Volume (Å^3)	296.644(7)	294.498(8)	291.877(6)	290.041
M–O (Å)	2.00895(2)	2.00297(2)	1.99673(2)	1.99106
M–Se (Å)	3.089(1)	3.082(1)	3.080(1)	3.07474
M–Se/*M*–O	1.5374(5)	1.5387(6)	1.5424(6)	1.54427
Se–Cu–Se (Å)	2.5104(4)	2.5059(7)	2.4949(6)	2.4916(6)
Se–Cu–Se, α^a (°) [2]	106.31(3)	106.13(4)	106.32(4)	106.09(6)
Se–Cu–Se, β^a (°) [4]	111.08(2)	111.17(2)	111.07(2)	111.19(3)

Appendix i. Alternate model for computation of crystal and electronic structures.

Another possible magnetic structure of $Sr_2NiO_2Cu_2Se_2$ is whereby alternate NiO_2 layers are exclusively high-spin or low-spin. This arrangement was achieved by first imposing different values of U_{Ni} in alternate layers (2 eV results in low-spin, 4 eV high spin) and then using the converged geometry and density to initialise a calculation where U_{Ni} = 2 eV was imposed for all metal centres. This mixed configuration offers remarkably good agreement with experiment – *c* is now underestimated by 0.26 Å and *a* by 0.03 Å, replicating precisely the behaviour of the Co/Cu/Zn systems. The Ni-O distances in the alternate layers are constrained to be the same by the symmetry of the lattice but the Ni-S distances now differ, with the high-spin (3.16 Å) and low-spin values (3.00 Å) values close to those computed for the all-high or all-low configurations, respectively.

References

(1) Warren, B. E. X-Ray Diffraction in Random Layer Lattices. *Phys. Rev.* **1941**, *59* (9), 693–698. https://doi.org/10.1103/physrev.59.693.

(2) Warren, B. E.; Bodenstein, P. The Shape of Two-Dimensional Carbon Black Reflections. *Acta Crystallogr.* **1966**, *20* (5), 602–605. https://doi.org/10.1107/s0365110x66001464.

(3) Zhao, L. L.; Wu, S.; Wang, J. K.; Hodges, J. P.; Broholm, C.; Morosan, E. Quasi-Two-Dimensional Noncollinear Magnetism in the Mott Insulator $Sr_2F_2Fe_2OS_2$. *Phys. Rev. B* **2013**, *87* (2). https://doi.org/10.1103/physrevb.87.020406.

(4) Smura, C. F.; Parker, D. R.; Zbiri, M.; Johnson, M. R.; Gál, Z. A.; Clarke, S. J. High-Spin Cobalt(II) Ions in Square Planar Coordination: Structures and Magnetism of the Oxysulfides $Sr_2CoO_2Cu_2S_2$ and $Ba_2CoO_2Cu_2S_2$ and Their Solid Solution. *J. Am. Chem. Soc.* **2011**, *133* (8), 2691–2705. https://doi.org/10.1021/ja109553u.

(5) Clarke, S. J.; Adamson, P.; Herkelrath, S. J. C. C.; Rutt, O. J.; Parker, D. R.; Pitcher, M. J.; Smura, C. F. Structures, Physical Properties, and Chemistry of Layered Oxychalcogenides and Oxypnictides. *Inorg. Chem.* **2008**, *47* (19), 8473–8486. https://doi.org/10.1021/ic8009964.

(6) Blandy, J. N.; Liu, S.; Smura, C. F.; Cassidy, S. J.; Woodruff, D. N.; McGrady, J. E.; Clarke, S. J. Synthesis, Structure, and Properties of the Layered Oxide Chalcogenides $Sr_2CuO_2Cu_2S_2$ and $Sr_2CuO_2Cu_2Se_2$. *Inorg. Chem.* **2018**, *57* (24), 15379–15388. https://doi.org/10.1021/acs.inorgchem.8b02698.

(7) Zhu, W. J.; Hor, P. H. Unusual Layered Transition-Metal Oxysulfides: $Sr_2Cu_2\mathit{M}O_2S_2$ (*M* =Mn, Zn). *J. Solid State Chem.* **1997**, *130* (2), 319–321. https://doi.org/10.1006/jssc.1997.7299.

(8) Broadley, S. *MChem*. Thesis, University of Oxford. **2005**.
